# Visualizing influenza A virus assembly by in situ cryo-electron tomography

**DOI:** 10.1038/s41467-025-65117-z

**Published:** 2025-10-23

**Authors:** Moritz Wachsmuth-Melm, Sarah Peterl, Aidan O’Riain, Jana Makroczyová, Konstantin Fischer, Tim Krischuns, Sílvia Vale-Costa, Maria João Amorim, Petr Chlanda

**Affiliations:** 1 Department of Infectious Diseases, Virology, Medical Faculty, Heidelberg University, Heidelberg, Germany; 2 BioQuant-Research Center for Quantitative Analysis of Molecular and Cellular Systems, Heidelberg University, Heidelberg, Germany; 3 Cell Biology of Viral Infection Lab (CBV), Católica Biomedical Research Centre (CBR), Católica Medical School — Universidade Católica Portuguesa, Lisboa, Portugal; 4 I3S-Instituto de Investigação e Inovação em Saúde, Universidade do Porto, Porto, Portugal

**Keywords:** Electron microscopy, Influenza virus

## Abstract

Influenza A virus (IAV) forms pleomorphic particles that package eight ribonucleoprotein complexes (vRNPs), each carrying a distinct RNA genome segment. vRNPs assemble in the nucleus and undergo selective sorting during Rab11a-mediated trafficking to the plasma membrane. Virion assembly is orchestrated by matrix protein 1 (M1), which forms a layer beneath the viral envelope containing hemagglutinin (HA) and neuraminidase (NA). However, molecular details of vRNP distribution, cytosolic trafficking, and coordination of IAV assembly remains unclear. Using in situ cryo-ET, we reveal that HA-containing membranes provide Rab11a-dependent platforms for membrane-assisted vRNP clustering, reducing inter-vRNP distances. In the absence of HA, vRNPs cluster on NA-containing membranes and virus assembly remains intact, indicating that vRNP clustering and trafficking is membrane-assisted but HA independent. The characteristic 7 + 1 vRNP bundle forms concomitantly with budding and is orchestrated by M1 layer assembly that precedes plasma membrane attachment. We further reveal that intracellular M1 forms multilayered helical assemblies of antiparallel dimers, structurally distinct from the M1 layer in virions. These assemblies are compact in the nucleus but partially dissociate in the cytoplasm, likely serving as a reservoir for budding. Together, our findings uncover membrane-assisted vRNP clustering and molecular details of M1 coordinated influenza virus assembly.

## Abstract (web summary)

Influenza A virus must package eight separate genomic segments, called viral ribonucleoproteins (vRNPs). Using in situ cryo-electron tomography, the authors visualize how vRNPs are clustered on cell membranes containing the viral glycoprotein hemagglutinin or neuraminidase, and how the matrix protein M1 forms intracellular structures that reorganize during budding to support packaging.

## Introduction

Influenza A virus (IAV) is a membrane-enveloped pleomorphic virus that incorporates a negative-sense single-stranded RNA genome, divided into 8 segments. Each genome segment is encapsidated into a distinct viral ribonucleoprotein complex (vRNP) composed of nucleoprotein (NP) forming a helical rod-shaped complex with RNA^1–3^. To produce an infectious progeny, virions incorporate one copy of all 8 segments in a specific 7 + 1 configuration where one central vRNP is surrounded by 7 vRNPs^4,5^. Virus RNA replication takes place in the nucleus of an infected cell^6,7^ where viral genome transcription utilizes cap-snatched primers^8^ and replication is driven by the tripartite polymerase complex composed of PA, PB1 and PB2 localized at the end of the vRNP. The polymerase forms a complex with complementary RNPs (cRNPs) encapsidating RNA of positive polarity serving as a template^9,10^. vRNP selective export depends on nuclear export protein (NEP) and chromosomal region maintenance 1 protein (CRM1)^11^. Upon export from the nucleus, vRNPs directly interact with Rab11a via the C-terminal domain of PB2^12^. This interaction was initially proposed as a mechanism for trafficking vRNPs to the plasma membrane, although it was immediately recognized that during infection Rab11a redistributed to form enlarged puncta that accumulated all vRNP types in the cytosol to be trafficked towards the plasma membrane^13–18^. Still, how and where during the trafficking vRNPs assemble into a 7 + 1-complex are among the longstanding questions in the influenza field. The answer to this question is pivotal to further understand molecular principles of IAV reassortment and zoonotic events. Multiple studies have used microscopy techniques to shed light on the mechanism underlying this process and several models have been proposed. Fluorescence In Situ Hybridization (FISH) and super-resolution microscopy studies have shown that vRNP clustering occurs en route to the budding site at the plasma membrane^19–21^. Recent studies revealed that vRNPs form liquid condensates that colocalize with Rab11a at endoplasmic reticulum (ER) exit sites^22^ and influenza A virus infection remodeled ER which facilitate the transport of vRNPs^23^. IAV surface glycoproteins hemagglutinin (HA) and neuraminidase (NA) undergo apical trafficking using Rab17 and Rab23 endosomal compartments^24,25^ and are targeted to cholesterol-rich lipid raft domains formed at the plasma membrane—the site of IAV budding^26,27^. This process is coordinated by matrix protein 1 (M1)^28^ that forms a helical matrix layer underneath the viral envelope^29,30^ and interacts with cytoplasmic tails of viral glycoproteins HA and NA^31^. Virus budding is concluded by membrane scission driven by the matrix protein 2 (M2) ion channel^32^. However, IAV cellular replication and assembly have not been investigated by cryo-electron tomography (cryo-ET) in near-to-native conditions. Due to the lack of context information during imaging in fluorescence microscopy and poor structural preservation of room temperature EM, single vRNPs have not yet been resolved in infected cells and thus the site of vRNP 7 + 1 bundle formation and their association with membranous or membrane-less organelles remains elusive. While it has been shown that the M1 layer interacts with vRNPs^32^, this process is not fully understood and has not yet been studied at molecular resolution in situ.

Here we applied cellular cryo-ET to study cells infected with either H1N1 or H3N2 IAV and were able to visualize single vRNPs forming clusters in both the nucleus and cytosol. Intriguingly, we uncovered that HA and NA form molecular arrays zippering endomembranes and discovered that vRNPs cluster on endomembranes containing HA arrays in a Rab11a-dependent manner. We show that vRNP density increases upon interaction with HA-membranes, thereby facilitating the probing of vRNP-vRNP interactions, which may lead to sorting. In the absence of HA, vRNPs are found associated with NA-membranes and infectious virions are assembled, indicating that vRNP clustering is membrane assisted but not HA dependent. Finally, our data uncover that M1 forms a multi-layered helical structure and assembles the viral matrix layer that closes prior to budding neck formation and drives vRNP incorporation into budding virions.

## Results and discussion

### Hemagglutinin and neuraminidase arrays induce membrane zippering in influenza A virus infected cells

**Fig. 1:**
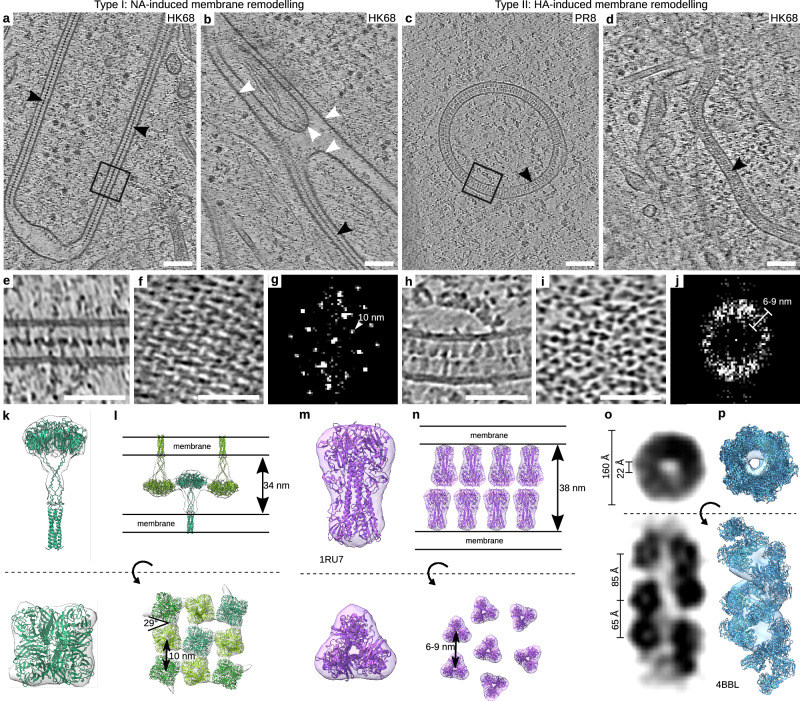
Influenza A virus glycoproteins form molecular arrays zippering host endomembranes. **a**–**d** Representative slices through cryo-electron tomograms of A549 cells infected with PR8 (22 grids) or HK68 (3 grids) at 16 h post-infection. HA and NA-membranes are indicated by black arrowheads, plasma membrane by white arrowheads. **e** Magnified slice through the tomogram of the NA-zipper shown in (**a**). **f** Representative slice of a tomogram showing the cross-section of the NA array. **g** Fast Fourier Transformation (FFT) of (**f**) indicating the NA-NA spacing of 10 nm. **h** Magnified slice through the tomogram of the HA-zipper shown in (**c**). **i** Representative slice of a tomogram showing the cross-section of the HA array. **j** FFT of (**i**) indicating the HA-HA spacing of 6 to 9 nm. **k**, **l** Subtomogram average of individual NA and a subtomogram average of NA arrays fitted with predicted NA structure delivered from AlphaFold2 with black lines indicating membrane bilayers. **m**, **n** Subtomogram average of HA with the fitted structure determined experimentally (PDB: 1RU7) and a model of the HA zipper with black lines indicating membrane bilayers. **o** Slice through the subtomogram average of vRNPs showing the major and minor groove. **p** Subtomogram average shown in (**o**) with the fitted experimentally determined vRNP structure (PDB: 4BBL). Subtomogram averages visualized with ChimeraX version 1.9^76^. Scale bars: (**a**–**d**) 100 nm; **e**, **f**, **h**, **i** 50 nm.

To investigate IAV replication and assembly, we performed in situ cryo-electron tomography (cryo-ET) of cryo-focused ion beam (cryo-FIB) milled A549 human lung epithelial cells infected with influenza A/Puerto Rico/8/1934 (H1N1) virus (hereafter, PR8), with a pandemic strain A/Hong Kong/1/68 (H3N2) (hereafter, HK68) or with A/WSN/1933 (H1N1) (hereafter WSN). At 16 h post-infection (hpi), we observed protein arrays in cellular remodeled endomembranes in all infected cells (Fig. 1a–d). Strikingly, we were able to detect two different types of membrane pairing, both resembling a zipper, forming membrane sheets and double membrane vesicles of variable sizes. Since zippers could be distinguished by structurally distinct luminal protein arrays obvious in the tomograms, we classified them as zipper type I (Fig. 1a, b; supplementary movie 1) and type II (Fig. 1c, d; supplementary movie 2). While type I was formed by a single layer with C4 symmetry and membrane-to-membrane distance of 34 nm (Fig. 1e), type II consisted of two parallel arrays with membrane-to-membrane distance of 38 nm (Fig. 1h). Type I zippers were found only in HK68-infected cells, whereas type II zippers were found in all cells infected with PR8 and HK68.

Subtomogram averaging (STA) revealed that type I zippers were formed by neuraminidase (NA) forming a crystalline array with lateral spacing of 10 nm (Fig. 1e–g) and head-to-head angular displacement of 29° (Fig. 1k, l; supplementary movie 3). Furthermore, we could demonstrate that type II zippers were formed by two opposing hemagglutinins (HA) in pre-fusion conformation (Fig. 1m, n). While the NA zipper was formed by NA-NA lateral interaction with alternating up and down NA headgroups, the HA zipper was formed by HA-HA ectodomain head-to-head interactions. Importantly, we could show that NA zippers can be connected to the plasma membrane (Fig. 1b), which suggests NA delivery to the plasma membrane in HK68 infected cells upon NA unzippering. Since NA zippers were not observed in PR8-infected cells, we sought to investigate possible structural differences between NA-PR8 (subtype 1) and NA-HK68 (subtype 2) in the NA-NA interacting regions. We used AlphaFold2^33^ to predict HK68 and PR8 NA structures and fitted them into the STA map (Supplementary Fig. 1k, l). The prediction of the NA tetrameric headgroup matched experimentally determined NA. A cryo-EM density at the membrane proximal site of the tetrameric headgroup contains numerous glycosylation sites, and this likely corresponds to multiple glycans. The structure predictions revealed differences in surface charge distribution between PR8 and HK68 NA which could explain the lack of NA-NA zippering in PR8 infection. To shed light on possible conservation across different N1 and N2 subtypes we investigated sequence similarities between different N1 and N2 IAV strains. The interacting loops of N1 are highly evolutionarily conserved from A/Puerto Rico/34 (H1N1) to recently reported H5N1 avian IAV reported in dairy cattle (A/Texas/37/2024 (H5N1)). In contrast, N2 contacting loops are less conserved but contain several conserved residues and loop 1 in N2 is highly negatively charged due to the presence of 2–3 aspartates. The net charge at pH 7.4 of PR8 loop 2 and HK68 loop 2 were calculated to be −2.663 and +1.151, respectively. This data indicates that there is an electrostatic interaction between HK68 loops 1 and 2 which is lacking in PR8 loops 1 and 2 (Supplementary Fig. 1).

**Fig. 2:**
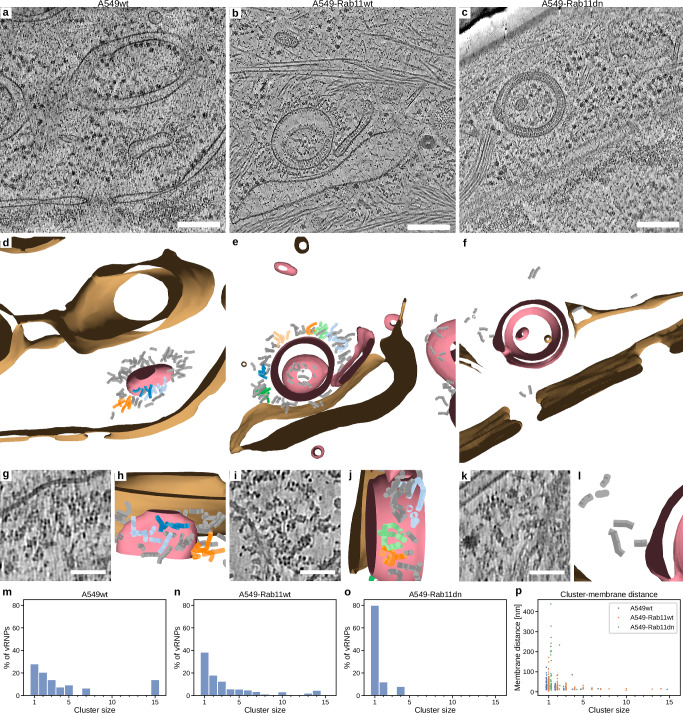
vRNPs cluster in the vicinity of HA-membranes in a Rab11a dependent manner. Representative slices through cryo-electron tomograms of an A549wt (**a**, 12 grids), A549-Rab11wt (**b**, 6 grids) and A549-Rab11dn (**c**, 4 grids) cell each showing an HA-membrane and vRNPs. **d**–**f** Segmentations of the tomograms shown in (**a**–**c**) depicting HA-membranes (pink), other membranes (brown) and vRNPs (various colors). vRNPs that are part of a cluster of one or two vRNPs are colored gray, while larger vRNP clusters are each assigned a different color. **g**–**l** Magnified views of the tomograms and segmentations shown in (**a**–**f**) displaying vRNP clusters on HA-membranes. Segmentations are rotated to better highlight vRNP clustering. **m**–**o** Distribution of vRNP cluster sizes in A549wt (**n**), A549-Rab11wt (**o**) and A549-Rab11dn (**p**) cells. **p** Shortest distance to HA-membranes for each vRNP cluster. All data plotted with seaborn version 0.13.2. Source data are provided as a Source Data file. Scale bars: **a**–**c** 200 nm; **g**, **i**, **k** 50 nm.

In contrast to the symmetric NA arrays, HA arrays showed HA-HA lateral spacing varying between 6 and 9 nm (Fig. 1i, j) which is consistent with HA-HA spacing on isolated virions^34,35^. HA-membranes were highly variable in size, with approximate diameters between 60 and 1220 nm (Supplementary Fig. 3a). Based on the extent of HA-zippering, we classified the HA-membranes into (i) single membranes coated with HA, (ii) double membranes with fully zippered HA and (iii) partial zippers displaying both phenotypes in the same membrane. 46% of observed HA-membranes were single membrane vesicles, whereas 28% were partially and 26% fully zippered double membranes that formed flat cisternae, autophagy-like compartments or double-membrane vesicles (Supplementary Fig. 3b). Occasionally, we observed that HA-membrane cisternae form joints resembling a  hemifusion state, indicating HA-membrane cisternae can undergo membrane fusion, leading to the formation of a closed compartment (Supplementary Fig. 2k, l). However, the limited thickness of cryo-lamellae prevents capturing entire HA-membranes, hindering quantitative assessment of their open or closed state. In contrast to NA, some HA-arrays were not found to form HA-HA head-to-head interactions, hence not all HA containing membranes formed an HA-zipper (Fig. 2a, b). Further, we also tested if HA-array formation and HA-HA membrane zippering is dependent on other viral factors such as M2, which was shown to modulate autophagy in IAV-infected cells^36^. To this end, we performed in situ cryo-ET on HEK-293T cells transfected with HA, which showed that HA expression alone is able to form arrays and induces membrane zippering indistinguishable from that observed in infected A549wt cells (Supplementary Fig. 2c). We next hypothesized that HA-HA zippering is induced by HA binding to sialic acid residues at the opposing HA. We therefore mutated Asn residues to Gln on the head domain of HA PR8 (N142Q, N144Q, N146Q, N206Q, N210Q, N212Q) but found no effect on HA zippering after ectopic expression in cryo-FIB milled cells. To test whether HA-zippering is also induced by evolutionarily and functionally distinct HA, HA bat subtype H18 (A/flat-faced bat/Peru/033/2010 (H18N11)) was ectopically expressed in VeroE6 cells. Unlike other HAs, H18 does not bind sialic acids but uses MHC-II as its receptor^37^. It shares only 50% sequence similarity with H1 (PR8) and 39% with H3 (HK68). Interestingly, our data show HA arrays and endomembrane zippering in cells transfected with H18 alone (Supplementary Fig. 2d). To investigate the origin of HA-containing endomembranes, we performed colocalization analysis between HA and the ER markers calnexin (CNX) and calreticulin (CLR), or ERGIC-53 (ER-Golgi intermediate compartment (ERGIC) marker) and GM130 (Golgi marker) at 4, 8 and 16 hpi in PR8 infected A549wt cells. These data show that at 4 hpi, HA primarily colocalizes with the Golgi, as well as with the ER and ERGIC. By 8 and 16 hpi, colocalization of HA with the Golgi and ERGIC decreases, likely due to ongoing remodeling of the Golgi apparatus, which disappears at 16 hpi, consistent with previously reported Golgi fragmentation^38^ (Supplementary Fig. 4a, b).

HA expression alone is not responsible for Golgi apparatus fragmentation (Supplementary Fig. 4c). Additionally, we could show that drug-induced Golgi apparatus disruption by H89^39^ and Brefeldin A^40^ does not change HA colocalization with ER and ERGIC or the overall distribution of the HA signal (Supplementary Fig. 4a, b). Overall, our data suggest that HA follows the canonical trafficking pathway through ER, ERGIC and Golgi. The strong colocalization with the Golgi early timepoints indicates that HA-membranes originate from the Golgi, which then gets fragmented and loses the GM130 marker as infection progresses.

### vRNPs interact with endomembranes containing HA arrays

On the cytoplasmic sides of HA-membranes, we regularly observed cylindrical densities with helical striation reminiscent of vRNPs (Fig. 1h; Supplementary movie 2). Subtomogram averaging of the densities confirmed the double-helical structure with major and minor grooves of 85 Å and 65 Å (Fig. 1o) which is consistent with the previously published structure of vRNPs from isolated virions (Fig. 1p)^2^. At 16 hpi, vRNPs formed variably dense accumulations associated with 74% (*n* = 109) of HA-membranes (Supplementary Fig. 3c) and only a single event was observed where vRNPs interacted with a vesicle not containing HA (Supplementary Fig. 2j). Interestingly, while vRNP accumulations were also observed at 8 hpi on 64% (*n* = 36) of HA-membranes, we observed 8 events of vRNPs interacting with non-HA containing membranes showing budding of coated vesicles that likely represent ER exit-sites (Supplementary Fig. 2i). This indicates that vRNPs accumulate on HA-membranes during later stages of infection. In the case of HA-zippered double membranes, vRNPs were found on both sides of the double membrane (Fig. 1c, h). A subset of vRNPs was directly in contact with HA-membranes, while vRNPs that were not directly interacting were found close to membrane-interacting vRNPs. Occasionally, vRNPs associated with HA-membranes or membranes lacking HA formed a zone devoid of any cellular material such as ribosomes, resembling a biomolecular condensate (Supplementary Fig. 2e, h; Supplementary movies 4, 5). Thus, our data agree with previously published studies^22,41–43^ showing NP signal in the vicinity of ER-exit sites. NA-zippers found in HK68 infected cells (*n* = 5) did not carry vRNPs besides one single example where vRNPs were found on a NA-zipper in a region devoid of NA (Supplementary Fig. 2f). Hence, our data demonstrate that HA-arrays containing endomembranes play a major, yet unexplored role in cytoplasmic vRNP transport and clustering.

### vRNP density is increased upon interaction with endomembranes containing HA-arrays in a Rab11a-dependent manner

The small GTPase Rab11a has been extensively demonstrated to be essential for all stages of cytoplasmic vRNP transport^13,14,44^. This suggests that Rab11a may also be important for vRNP delivery to HA-membranes, or that HA-membranes themselves are the trafficking organelle delivering vRNPs to the plasma membrane. To test this hypothesis, we performed in situ cryo-ET on PR8-infected A549 cells constitutively overexpressing Rab11a wild-type (A549-Rab11wt) or Rab11a dominant-negative S25N mutant (hereafter, A549-Rab11dn) fused to green fluorescent protein (GFP)^45^. Both cell lines displayed HA array containing endomembranes like A549wt cells (Fig. 2a–c), with no significant differences in sizes of HA-membranes (Supplementary Fig. 3a). Notably, we observed a reduced association of vRNPs with HA-membranes in A549-Rab11dn cells. In contrast to A549wt and A549-Rab11wt cells where 77% and 90% of analyzed HA-membranes carried vRNPs, respectively, we observed only 40% of HA-membranes with vRNPs in A549-Rab11dn cells (Supplementary Fig. 3c). In addition, the number and density of vRNPs apparent in the tomograms were lower in A549-Rab11dn cells. Importantly, both viral RNA and protein levels were the same in PR8-infected A549wt, A549-Rab11wt and A549-Rab11dn at 4, 8, 12, and 16 hpi (Supplementary Fig. 5) but A549-Rab11dn produced fewer infectious particles^18^.

To gain insights into vRNP-vRNP interactions and their spatial distribution on HA-membranes, individual vRNPs were segmented and subjected to nearest-neighbor analysis (Fig. 2d–i). This allowed close inspection of vRNP-vRNP interactions in the context of membrane binding and quantitative assessment of the degree of clustering.

Because the fraction of HA-zippered double membranes was increased from 28 to 53% in A549-Rab11dn cells when compared to A549wt cells (Supplementary Fig. 3b), we aimed to analyze whether HA-zippering affects the HA-membrane-vRNP interaction. The vRNP spatial analysis shown in Supplementary Fig. 3d shows no significant difference in vRNP-membrane distances between zippered and single HA-membranes (*p* = 0.13). Overall, our data show that vRNPs interact with HA-membranes in Rab11a dependent but HA-HA zippering independent manner.

The vRNP spatial analysis confirmed a significant increase in vRNP-vRNP distances in A549-Rab11dn cells compared to A549wt (*p* = 4.9e-07) and A549-Rab11wt (*p* = 9.8e-08) cells, even though the vRNP-membrane distances are only significantly different between A549-Rab11dn and -Rab11wt cells (*p* = 1.7e-3) (Supplementary Fig. 3e, f). These differences prompted us to investigate the clustering of vRNPs in more detail and validate clustering against a model with randomly distributed vRNPs. To this end, vRNP clusters were identified based on vicinity using a center-to-center threshold of 18 nm. This revealed vRNP clusters of up to 15 vRNPs on HA-membranes in A549wt and A549-Rab11wt cells (Fig. 2m, n) in contrast to only 4 vRNPs in A549-Rab11dn cells (Fig. 2o). Furthermore, increasing cluster size correlated with a shorter distance to HA-membranes (Fig. 2p). Clustering was reduced in a random vRNP model, independent of the vicinity threshold (Supplementary Fig. 3h, i). Overall, this data show that HA-membranes serve as a platform that increases vRNP density at the HA-membrane surface.

To assess whether Rab11a associates with HA-positive membranes, we performed cryo-correlative light and electron microscopy (cryo-CLEM) to localize GFP-Rab11a directly on cryo-FIB-milled lamellae. This approach allowed us to target cryo-ET data acquisition to regions enriched in GFP-Rab11a. Of the 21 tomograms acquired at GFP-Rab11a–positive sites, 86% contained HA-positive membranes (Supplementary Fig. 6a–d), indicating that Rab11a is frequently found in the vicinity of HA-membranes.

To further investigate Rab11a localization, we performed immunofluorescence confocal microscopy on PR8 infected A549-Rab11wt cells stained for HA and PB2—the latter serving as a marker for vRNPs and a known Rab11a interaction partner (Supplementary Fig. 6e–i)^12^. Consistent with previous reports, we observed strong colocalization between PB2 and Rab11a in the cytoplasm (Supplementary Fig. 6j). In addition, we observed low colocalization between HA and Rab11a and moderate colocalization between HA and PB2. This indicates that there is no direct interaction between Rab11 and HA, consistent with a previous study^25^.

Together, these data indicate that Rab11a localizes near HA-positive membranes and likely facilitates the handoff of vRNPs for further assembly or trafficking at these sites.

### In the absence of HA, vRNP clustering takes place on NA-containing membranes

**Fig. 3:**
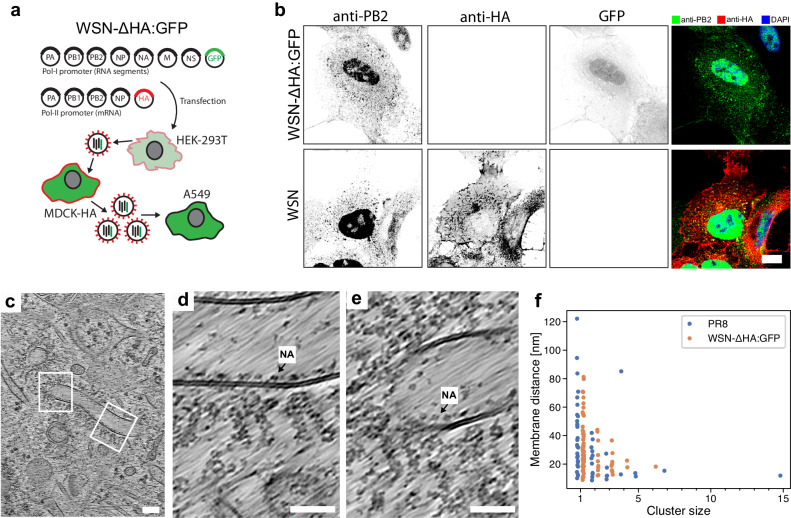
vRNP interactions in the absence of HA in WSN-ΔHA:GFP virus infection. **a** Schematic of a 12-plasmid reverse genetics system to generate WSN-ΔHA:GFP virus, which encodes for GFP instead of HA and can replicate only in transcomplementing HA-expressing cells. In A549 cells it is limited to a single round of infection. **b** Confocal microscopy slices of A549 cells infected either with WSN or WSN-ΔHA:GFP virus fixed at 16 hpi and immunostained with the indicated antibodies. 10 cells analyzed. Scale bar: 10 µm. **c**–**e** Slices of cryo-electron tomograms capturing NA-containing organelles associated with vRNPs. NA with tetrameric headgroup is highlighted. A total of 6 NA membranes with vRNPs was observed in 4 cells. Scale bars: 100 nm (**c**), 50 nm (**d**, **e**). **f** Distributions of vRNP cluster sizes determined by nearest-neighbor vRNP-vRNP analysis on NA (WSN-ΔHA:GFP) or HA (PR8) containing organelles in A549wt cells. There is no significant difference in the distribution of cluster sizes (*p* = 0.33) or their distances to membranes (*p* = 0.16) according to a two-sided two-sample Kolmogorov–Smirnov test. All data plotted with seaborn version 0.13.2. Source data are provided as a Source Data file.

Previous studies provide conflicting results whether HA is required for vRNP bundle assembly at the plasma membrane^46,47^. To further elucidate the function of HA in vRNP trafficking, we generated a recombinant A/WSN/33 (H1N1) (hereafter WSN) virus that encodes for a GFP segment instead of the HA segment, referred to as WSN-ΔHA:GFP. This virus can only replicate in MDCK cells constitutively expressing HA (MDCK-HA) and is restricted to a single round of infection in the absence of in-trans HA expression (Fig. 3a, b). Wild-type WSN virus showed HA-remodeled organelles interacting with vRNPs (Supplementary Fig. 2m).

Notably, in situ cryo-ET of A549 cells infected with WSN-ΔHA:GFP revealed that vRNPs bind to NA-containing membranes (Fig. 3c–e). Considering that HA and NA cytoplasmic tails have different amino acid sequences, this data indicate that vRNP do not specifically interact with HA or NA but rather either HA or NA traffic together with vRNPs towards the plasma membrane. Moreover, these NA-containing membranes were not zippered, ruling out the possibility that membrane zippering is crucial for vRNP trafficking. Spatial analysis of vRNP-vRNP interactions showed no differences in vRNP clustering in the presence or absence of HA (Fig. 3f). Importantly, in both cases vRNP clustering is increased at the membrane surface (Fig. 3f).

### M1 forms cylindrical assemblies in the nucleus and cytoplasm

**Fig. 4:**
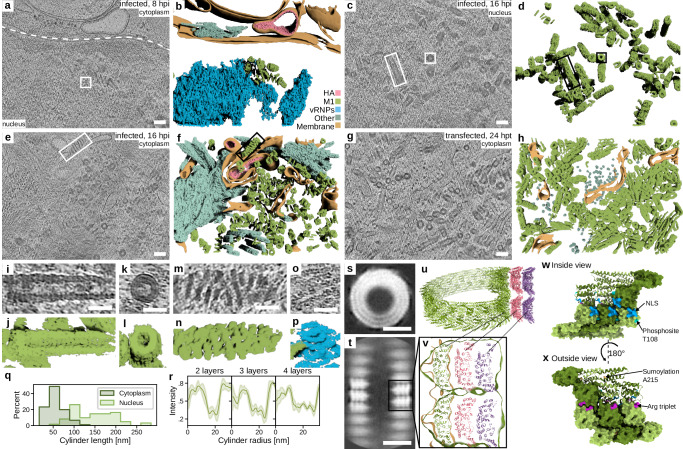
M1 forms cylindrical assemblies in the nucleus that disassemble in the cytoplasm. Representative slices through cryo-electron tomograms of A549wt cells infected with PR8 at 8 (**a**, 2 grids) and at 16 (**c**, **e**, 22 grids) hours post-infection showing a cluster of compact M1 cylindrical assemblies in the nucleus (**c**) and mixed loosely coiled and compact assemblies inside the cytoplasm (**e**). The nuclear envelope is depicted with a white dashed line in (**a**). **b**, **d**, **f**, **h** Segmentations of the tomograms shown in (**a**, **c**, **e**, **g**). Legend is shown in (**b**). **g** Representative slice through a cryo-electron tomogram of a VeroE6 cell transfected with M1 (HK68) at 24 h post-transfection and its segmentation in (**h**) (2 grids). **i**–**p** Magnified areas of tomograms and segmentations indicated by white rectangles in (**a**, **c**, **e**) showing vRNPs interacting with M1 (**o**, **p**), top and side views of condensed M1 cylinders (**i**–**l**) and a side view of a loosely coiled M1 cylinder (**m**, **n**). **q** Lengths of condensed M1 cylinders in nucleoplasm (light green) and cytoplasm (dark green), *p* = 8.7e-32 according to two-sided T-test. **r** Radial profiles of condensed M1 cylinders of different diameters revealing different numbers of layers. Error bars indicate the 95% confidence interval. Top (**s**) and side (**t**) views of a helical subtomogram average of nuclear assembled M1 helices. Average calculated from 17 tomograms **u**, **v** Model based on M1 helical structure PDB:6Z5L fitted into a single helical layer of the M1 cylinder. **u** A stack of three M1 layers (green, red, and magenta) based on PDB:6Z5L of M1 in multi-layered M1 helices. **v** Overlay of cryo-EM map EMD-11079 (gold contour), M1 atomic model (green, red, magenta), a cross section of two helical turns of M1 cylinder subtomogram average (green contour) shown in (**t**). **w**, **x** Inside and outside views of the M1-M1 antiparallel arrangement of a single layer in PDB:6Z5L with indicated arginine triplet, nuclear localization signal (NLS), sumoylation and phosphorylation sites. All data plotted with seaborn version 0.13.2. Subtomogram averages visualized with ChimeraX version 1.9^76^. Source data are provided as a Source Data file. Scale bars: **a**, **c**, **e**, **g** 100 nm; **i**, **k**, **m**, **o** 50 nm; **s**, **t** 25 nm.

In the current model of the IAV replication cycle, vRNPs are assembled in the nucleus of infected cells and exported to the cytoplasm in a complex with nuclear export protein (NEP) and M1^48^. In agreement with this model, we resolved a cluster of vRNPs in the nucleus of an A549 cell infected with PR8 at 8 hpi (Fig. 4a, b, o, p; supplementary movie 6), confirming that vRNPs are fully assembled in the nucleus. In addition to vRNPs, our data on the nuclei of both PR8 and HK68 infected cells revealed hollow cylinders (Fig. 4c, d, i–l) with lengths between 65 and 285 nm (Fig. 4q) and inner diameters of 21 nm. Their outer diameter was highly variable, between 47 and 61 nm. Radial averaging of cylinder top views revealed 2 to 4 layers with a spacing of approximately 7 nm (Fig. 4r). These assemblies structurally correspond to previously reported M1 multilayered helically ordered structures which form in vitro from purified M1 protein at high salt concentration^29^. To confirm their identity in cells, we employed in situ cryo-ET on VeroE6 cells ectopically expressing M1 (HK68) alone, which revealed multilayered hollow cylinders in the nucleus and cytoplasm (Fig. 4g, h). To obtain more details on the arrangement of M1 in the helical assemblies, we performed STA on nuclear M1 assemblies in PR8 infected cells. The resulting map showed that the M1 cylinder is composed of helically arranged congruently stacked layers. Interestingly, the dimensions and the shape of individual layers within the M1 cylinder match the single-layered M1 helical tube formed in vitro in the presence of a nucleic acid and was determined by cryo-EM (PDB: 6Z5L)^30^. This tube consists of helical coils formed by M1 antiparallel dimers. Our STA map fits well to the M1-M1 antiparallel dimers including a density corresponding to nucleic acids in the proximity of the positively charged, Arg triplet containing helix 6 at the N-terminal domain (NTD) but it does not contain a nucleic acid density at the lateral layer-layer C-terminal domain interface (Fig. 4s–v). Similar M1 cylindrical structures were also found inside virions upon acidification as an M1 layer disassembly product^49^ and in cells infected by 2009 pandemic strain A/Lyon/969/09 (H1N1)^50^. Collectively, this shows that M1 forms helically organized multi-layered cylinders that are present in cells infected by different IAV strains. Based on the measured nuclear pore complex (NPC) diameter in infected cells (110–155 nm), the M1 cylindrical assemblies can pass through the NPC. This is further supported by our data showing M1 cylindrical assemblies in the cytoplasm in the vicinity of HA-membranes (Fig. 4e, f, m, n; supplementary movie 7) and close to viral budding sites (Supplementary Fig. 7a–e). Interestingly, cytoplasmic M1 assemblies were significantly shorter (Fig. 4q) and more often loosely coiled (nucleus: 8%; cytoplasm: 31%; *n* = 197; *p* = 1.9e-4). This indicates that M1 forms cylindrical assemblies in the nucleus that upon export to the cytoplasm become unstable and disassemble. Alternatively, M1 cytosolic cylinders are assembly intermediates and cylinder assembly is completed in the nucleus. Notably, transfection of M1 alone leads to formation of M1 multilayered helical assemblies (Fig. 4g, h; Supplementary Fig. 7l). Barnard’s exact test showed no significant difference in the fraction of nuclear and cytoplasmic cylinders between infected and transfected cells (nucleus infected 34%; nucleus transfected 41%; *p* = 0.07; *n* = 736). Similarly, there was no significant difference in condensed-to-loose cylinder ratio between infected and transfected cells when considering only the nucleus (infected 92%; transfected 97%; *p* = 0.08; *n* = 287). However, the condensed-to-loose cylinder ratio in the cytoplasm of transfected cells is significantly higher than in infected cells (infected 69%; transfected 100%; *p* = 1.1e-23; *n* = 449). This indicates that a viral component is needed for the loose conformation of M1 cylinders in the cytosol and that M1 cylinders might be utilized for virus assembly.

M1 cylinder disassembly could be regulated by another viral protein or by post-translational modifications such as phosphorylation (T108), ubiquitination and sumoylation (A215) that have been reported to regulate IAV assembly^51,52^. The phosphosite T108 is localized at the vicinity of nuclear localization signal (NLS) and could regulate M1 layering while the sumoylation site (A215) is positioned at the C-terminal domain (CTD) and could control M1 oligomerization and transition between M1 cylinder and M1 layer (Fig. 4w, x). Multilayered M1 cylinders were shown to be stabilized by hydrophobic interactions between M1 N-terminal domains (NTD)^29^. In addition, we propose that the M1-M1 electrostatic interactions engage the positively charged arginine triplet and nucleic acids or anions at the NTD (Fig. 4x). These NTD-NTD interactions are likely disrupted by M1 recruitment to the plasma membrane by M2^53^, where interactions with the glycoproteins^54^ and negatively charged phospholipids^55,56^ stabilize a single-layer M1 matrix in the budding virions^30^. We propose that the transition from M1 cylinder to M1 layer is the inverse process to matrix layer disruption during virus entry at endosomal pH, and M1 delivers a large amount of building blocks to the plasma membrane to aid virus budding.

### M1 layer formation drives the incorporation of vRNPs into the budding virions in an HA-independent manner

**Fig. 5:**
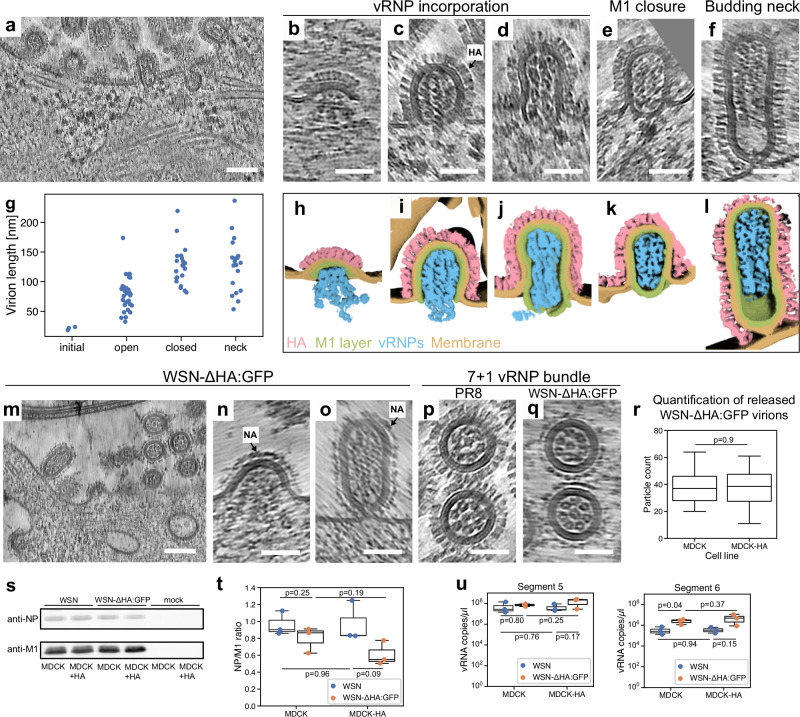
M1 layer formation drives incorporation of vRNPs into the budding virions. **a** Slice through a tomogram of an A549wt cell infected with PR8 at 16 hpi. Numerous viruses are budding or have already successfully budded. Representative slices through tomograms of budding viruses at different stages of budding, from initial (**b**) over open M1 layer (**c**, **d**), closed M1 layer (**e**), budding neck (**f**). **g** Quantification of protruding PR8 virion lengths during budding per stage of budding. 74 budding particles from 8 grids were analyzed. **h**–**l** Segmentations of the areas shown in (**b**–**f**). Scale bars: **a** 100 nm; **b**–**f** 50 nm. **m**–**o** Slice through a tomogram of A549wt cell infected with WSN-ΔHA:GFP at 16 hpi. NA is highlighted by arrows. Budding events were observed on 2 grids. **p** Slice through a tomogram of PR8 virus showing 7 + 1 vRNP bundle. **q** Slice through a tomogram of WSN-ΔHA:GFP virus showing 7 + 1 vRNP bundle. **r** Comparison of WSN and WSN-ΔHA:GFP virion release into the supernatant determined by cryo-EM in an mapping area of 62304 µm². 20 maps of WSN and 15 maps of WSN-ΔHA:GFP particles were analyzed. **s** Western Blot analysis of M1 and nucleoprotein (NP) release into the supernatant in WSN infected MDCK or MDCK stably expressing HA (MDCK-HA), and in WSN-ΔHA:GFP infected MDCK or MDCK stably expressing HA (MDCK-HA). Data from three independent experiments. **t** Bar plot showing NP:M1 release ratio. Data from three independent experiments. **u** RT-PCR quantification of released segment 5 and 6 upon infection of MDCK or MDCK stably expressing HA (MDCK-HA), and in WSN-ΔHA:GFP infected MDCK or MDCK stably expressing HA (MDCK-HA). All data plotted with seaborn version 0.13.2. Boxes represent the first and third quartiles, whiskers extend to points within 1.5 interquartile ranges. Indicated *p*-values were calculated using a two-sided T-test in scipy version 1.15.3. Source data are provided as a Source Data file. Scale bars: **a**, **m** 100 nm; **b**–**f**, **n**–**q** 50 nm.

To further shed light on the vRNP bundling process and the role of M1, we investigated IAV budding profiles by whole cell cryo-ET and cryo-ET performed on cryo-FIB milled lamellae. Multiple stages of PR8 budding were observed (Fig. 5a, g): initial membrane bending induced by a small patch of a spherically curved M1 layer (4%, Fig. 5b, h); budding virions with incorporated vRNPs with open M1 layer (42%, Fig. 5c, d, i, j); closed or partially closed M1 layer (28%, Fig. 5e, k), fully assembled with budding neck (26%, Fig. 5f, i) and released viruses showing 7 + 1 vRNP bundle (not quantified, Fig. 5p). Importantly, cryo-ET allowed us to advance the mechanistic understanding of the budding process. In the virions with a partially closed M1 layer, the M1 layer was not fully attached to the membrane in the virions and had free curved ends interacting with vRNPs (Fig. 5d, j; Supplementary Fig. 7h, i). This suggests that the M1 layer assembly precedes its attachment to the plasma membrane and M1 thereby drives the incorporation of vRNPs into the budding spherical (Fig. 5) and short filamentous PR8 virions (Supplementary Fig. 7h, j). In contrast, HK68 long filamentous virions captured at the end of the budding process contained a small gap in the M1 layer in the trailing tip of the particle in 47% cases (*n* = 17; Supplementary Fig. 7f, g). This gap in the M1 layer at the trailing end is observed significantly less often in budding PR8 virions (4%; *n* = 55; *p* = 2.6e-05). Overall, this highlights an important difference in the termination of budding in long filamentous HK68 and spherical PR8 virions. The M1 layer in spherical PR8 viruses likely interacts stronger with vRNPs (Supplementary Fig. 7h, i) and preferentially forms spherical helices rather than cylindrical helices which are typical for HK68 budding virions. Interestingly, we found that WSN-ΔHA:GFP virus lacking HA is able to assemble and incorporate vRNPs in 7 + 1 configuration (Fig. 5m, n, o, q). Cryo-EM of released WSN-ΔHA:GFP virions as well as Western blot analysis of M1 and NP proteins revealed that WSN-ΔHA:GFP showed similar release efficiency as WSN (Fig. 5r–t). Finally, incorporation of segment 5 and 6 was not different in WSN-ΔHA:GFP virions when compared to WSN, confirming that HA is dispensable for vRNP trafficking and clustering (Fig. 5u).

It is noteworthy that vRNP bundles reminiscent of a 7 + 1 parallel configuration^4,5^ were found neither on HA-membranes, nor NA-membranes (WSN-ΔHA:GFP infection) nor on the plasma membrane, but only in fully assembled virions. Hence, we propose that vRNP clustering on HA-membranes (or NA-membranes in the absence of HA) leads to sorting vRNPs consistent with studies showing that vRNP sorting occurs en route to the plasma membrane^19,20^. Sorted vRNPs subsequently assemble into a parallel 7 + 1 bundle upon contact with a patch of M1 layer at the plasma membrane, presumably as a result of energetically most favorable packaging geometry within the M1 helically organized scaffold.

**Fig. 6:**
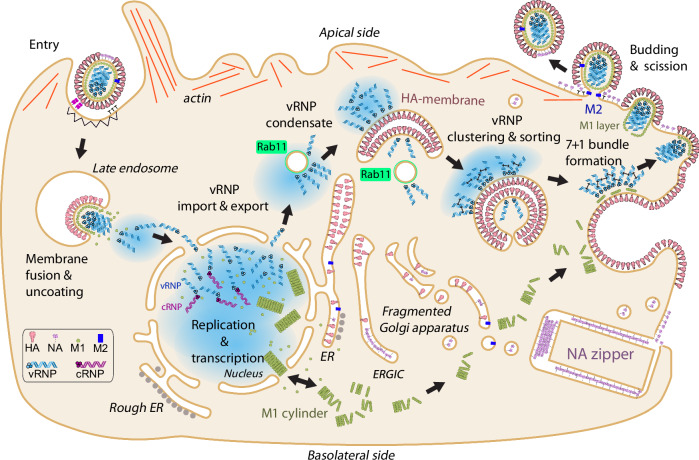
Model of IAV infection and vRNP trafficking summarizing findings in this study. IAV enters by endocytosis. After HA-mediated membrane fusion, vRNPs are imported to the nucleus for replication. IAV replication and transcription take place in the nucleus. vRNPs and complementary RNPs (cRNPs) are assembled in the nucleus and vRNPs are exported to the cytoplasm where they form a condensate which interacts with HA-membranes. HA-membranes are derived from ER and ERGIC and can be remodeled by HA-HA zippering. HA-membranes serve as clustering platforms in a Rab11-dependent manner and allow for vRNP sorting (indicated by small black double arrows). HA-membranes are trafficked to the plasma membrane where they fuse, delivering vRNPs and HA to budding sites. M1 cylinders disassemble in the cytoplasm delivering the building blocks for the formation of the M1 layer, which drives the incorporation of vRNPs into budding virions and membrane scission is mediated by M2 protein. We propose that the cytoplasmic domain (CTD) of the M1 protein is sterically concealed within the M1 cylinder, hence it cannot interact with vRNPs. M1 cylinders disassemble in the cytosol and M1 interacts with HA and M2 cytosolic tails at the plasma membrane which leads to the formation of the M1 layer and exposure of CTD that is available for vRNP interaction. The M1 layer closes, driving the formation of a budding neck. Infection by some IAV strains (H3N2 but not H1N1) leads to formation of NA zippers, where NA forms highly ordered 2D crystals zippering endomembranes that can fuse with the plasma membrane to deliver NA. In N1 strains, NA is delivered to the plasma membrane as single glycoproteins.

In summary, the data presented here allowed us to uncover the interplay of several viral proteins during IAV late replication stages and propose a model (Fig. 6). We identified HA as a mediator of membrane remodeling, which provides a platform to probe vRNP-vRNP interactions in a Rab11a dependent manner. This provides a large membrane surface that could allow for vRNP sorting and clustering as previously determined by FISH and super-resolution microscopy^20,22^. This work reconciles previous contradicting models showing that vRNPs interact with membranes^23^ but engage in vRNP-vRNP interactions to form membrane-associated biomolecular condensates^22^.

Interestingly, we show that HA compartments occasionally form closed double-membrane vesicles entrapping vRNPs (Fig. 2; Supplementary Fig. 2a, b). This indicates that a fraction of vRNPs is released from the cell upon fusion of the double-membrane vesicle with the plasma membrane. Finally, we report that M1 assembles into multilayered helical structures in the nucleus that disassemble in the cytoplasm, leading to the formation of the M1 layer, thus unraveling a potential pan-flu antiviral avenue by targeting the M1 cylinder. Overall, this data highlights the multifunctional role of the M1 protein and uncovers the existence of the M1 cylindrical assemblies in infected cells, which can also be formed upon M1 transfection.

Based on these findings, we propose the following model: Rab11a delivers vRNPs to HA-enriched membranes. The large surface area of the membrane compartment likely provides an interacting platform which facilitates vRNP clustering and sorting. vRNP clustering is HA-independent and hence whether HA and vRNP specifically interact remains to be elucidated. However, our data indicate that HA and vRNP interaction is indirect. To prevent premature virus assembly during vRNP transport, M1 is trafficked independently in the form of cylindrical structures. After reaching the plasma membrane, M1 interacts with M2 and negatively charged lipids, triggering a conformational change that exposes the vRNP-binding domain of M1. Upon subsequent interaction with the cytoplasmic tails of HA (or alternatively NA in WSN-ΔHA:GFP virus infection), M1 oligomerizes into a helical layer, which is essential for virus assembly. This helical M1 scaffold provides the necessary interface for vRNP incorporation via the M1 C-terminal domain.

## Methods

### Cells

A549^57^, obtained from American Type Culture Collection (ATCC), A549-Rab11KO^18^ (kindly provided by Prof. Balaji Manicassami, University of Iowa, USA) A549-GFP-Rab11wt and A549-GFP-Rab11dn (kindly provided by Prof. Maria João Amorim, Instituto Gulbenkian de Ciência, Portugal) cells were maintained in DMEM/F-12-GlutaMAX™ (Gibco, 31331-093), HEK-293T (ATCC) and MDCK (Prof. Maria João Amorim) cells in DMEM-GlutaMAX™ (Gibco, 61965-059), both supplemented with 100 U/ml penicillin-streptomycin (P/S) and 10% (v/v) fetal bovine serum (FBS). Cells were subcultured twice per week. All incubation steps were performed at 37 °C and 5% CO₂ unless otherwise noted.

### Reverse genetics

Influenza A/Puerto Rico/8/1934 (H1N1) and A/Hong Kong/1968 (H3N2) were rescued using an 8-plasmid reverse genetics system using pHW2000 bidirectional vectors. 2.7 × 10⁶ HEK-293T cells were seeded in a 10 cm cell culture dish in 15 ml complete DMEM and incubated for 24 h. Cells were then transfected with 8 plasmids, each corresponding to one genome segment. To this end, a transfection mix was prepared by mixing 1.5 ml OptiMEM with 1.875 μg of each plasmid (15 μg DNA in total) and adding 45 μl TransIT-LT1. The transfection mix was incubated for 20 min and carefully added on top of the cells. The cells were incubated for 24 h. Next, the medium was exchanged with 10 ml serum-free DMEM supplemented with 100 U/ml P/S, 1 μg/ml TPCK-trypsin and 0.3% BSA and 4 × 10⁶ MDCK cells were seeded into the same cell culture dish. After 24 h of incubation, the supernatant was harvested and centrifuged at 1000 × *g* for 10 min to remove cell debris. The cleared supernatant was snap-frozen in liquid nitrogen and used as p0 virus stock for virus propagation.

A/WSN/1933 (H1N1) viruses were rescued using a 12-plasmid reverse genetics system using RNA expression vectors encoding all eight viral segments and protein expression vectors encoding PA, PB1, PB2 and NP. To generate a A/WSN/1933:GFP virus lacking HA (WSN-ΔHA:GFP), vectors encoding seven RNA segments (PA, PB1, PB2, NP, NS, NA and M) plus a segment encoding GFP with HA packaging signals^58^ and protein expression vectors encoding PA, PB1, PB2, NP, and HA were used. Rescue was performed as described by Sjaastad et al.^59^ using HEK-293T cells and MDCK cells stably expressing HA (as described by Spieler et al.^60^).

### Virus propagation

MDCK cells were grown in a T175 flask to confluency. P0 virus stock prepared above was thawed on ice and diluted in 10 ml DMEM supplemented with 100 U/ml P/S. MDCK cells were washed twice with PBS. The diluted virus was added to the cells and spread evenly by tilting the plate. Cells were incubated for 1 h to allow virus uptake. Then, the cells were washed with infection medium (DMEM, 100 U/ml P/S, 1 μg/ml TPCK-trypsin and 0.3% BSA) and incubated in 40 ml infection medium. Cells were checked daily to watch for onset of cytopathic effect. Once most cells were detached, the supernatant was harvested, centrifuged at 1000 × g for 10 min, aliquoted and snap-frozen in liquid nitrogen.

### Virus titration by plaque assay

Into each well of two 6-well plates, 1 × 10⁶ MDCK cells were seeded in 2 ml complete DMEM (100 U/ml P/S, 10% FBS) and incubated for 24 h. The virus supernatant was thawed on ice and serially diluted from 10⁻³ to 10⁻⁸ in 10-fold steps in serum-free DMEM. Cells were washed twice with PBS, then 900 μl virus dilution was added to each well such that each dilution was titrated in duplicates. After incubation for 1 h, cells in each well were washed twice with PBS and overlaid with 3 ml DMEM supplemented with 1.2% Avicel-RC581, 0.14% BSA, 100 U/ml P/S and 1 μg/ml TPCK-trypsin. PR8 infected cells were incubated for two days, HK68 infected cells were incubated for four days to allow plaque formation.

To count plaques, the overlay medium was aspirated and cells were washed twice with PBS. Cells were chemically fixed for 30 min with 1 ml 1% glutaraldehyde in PBS per well. Each well was washed with PBS, then 200 μl crystal violet was added to each well. After 10 min, each well was washed twice with water to reveal the plaques. For each well, the titer was calculated using the formula $T=\frac{{n}_{{\mathrm{plaques}}}}{900\,{\mathrm{\mu l}}\times {\mathrm{dilution}}}$. The final titer used in experiments was the mean of all titers from wells with a quantifiable number of plaques.

### Immunofluorescence microscopy

5 × 10⁴ A549wt or A549-Rab11wt cells were seeded onto 12 mm microscopy coverslips (No. 1.5, Marienfeld) in a 24-well plate in 500 μl complete growth medium (DMEM/F-12, 10% FBS, 100 U/ml P/S) and incubated for 24 h. For infection experiments, cells were washed twice with PBS and infected with PR8 or HK68 virus, respectively, diluted in FBS-free DMEM/F-12 with an MOI of 3 for 1 h. Then, cells were washed twice with PBS and incubated in complete DMEM/F-12 for 16 h. In case of brefeldin A or H89 treatment, the medium was supplemented with 1× brefeldin A (eBioscience) or 20 μM H89 (InSolution). For transfection experiments, a transfection mix was prepared by mixing 50 μl OptiMEM with 0.5 μg pCAGGS-HA(PR8) plasmid and adding 1.5 μl Trans-IT LT1. The transfection mix was incubated for 20 min, then carefully added on top of the cells. The cells were then incubated for 24 h. After incubation, cells were washed twice with PBS and fixed with 4% paraformaldehyde (PFA) in PBS for 15 min. Then, cells were washed twice with PBS and permeabilized with 0.2% Triton-X100 in PBS for 7 min. For blocking, cells were washed twice with PBS and incubated with blocking buffer (3% BSA, 0.1% Tween-20 in PBS) for 30 min. Then, the cells were washed twice with dilution buffer (1% BSA, 0.1% Tween-20 in PBS) and incubated with the primary antibodies (mouse anti-HA stalk-binding antibody MEDI8852 1:500; rabbit anti-PB2 GTX125926 lot no. 44902 1:1000; rabbit anti-Rab11a 2413S 1:50; rabbit anti-calreticulin SPC-122 1:100; rabbit anti-calnexin 2679S 1:100; rabbit anti-ERGIC-53 13364-1-AP lot no. 00114367 1:500; rabbit anti-GM130 12480S lot no. 4 1:100) in dilution buffer for 1 hour. The cells were washed twice with dilution buffer and incubated with the secondary antibodies (Invitrogen A11034 Alexa Fluor 488 goat anti-rabbit lot no. 2110499 1:1000; Invitrogen A11030 Alexa Fluor 546 goat anti-mouse lot no. 2026145 1:1000; Invitrogen A11010 Alexa Fluor 546 goat anti-rabbit lot no. 2189179 1:1000; Invitrogen A21052 Alexa Fluor 633 goat anti-mouse lot no. 2126815 1:1000) and 300 nM 4’,6-diamidino-2-phenylindole (DAPI) in dilution buffer for 1 h. Before mounting, cells were washed twice with PBS and once with water. The coverslips were then briefly blotted on the side and placed upside down on a 7 μl drop of Prolong Glass Antifade Mountant on a microscopy slide.

Confocal microscopy of infected and transfected A549wt and A549-Rab11wt cells was performed on a Zeiss Airyscan 2 LSM 900 laser scanning confocal microscope equipped with a Zeiss Plan-Apochromat 63×/1.4 Oil immersion objective. Stacks were acquired with a pixel spacing of 71 × 71 × 130 nm and post-processed using Zeiss ZEN 3.2 (blue edition) Airyscan processing.

Pearson’s colocalization coefficients were calculated using SciPy version 1.15.3. In all colocalization analyses were restricted to the cytoplasm using a binary mask generated using a fixed threshold for the DAPI channel.

### Plunge freezing of infected and transfected cells

22 × 10⁴ A549, A549-Rab11wt, A549-Rab11dn, A549-Rab11KO, VeroE6 or HEK-293T cells were seeded onto Quantifoil R1.2/20 200 mesh grids in a 35 mm cell culture dish coated with SYLGARD™ 184 silicone elastomer. Both grids and dishes were glow-discharged and disinfected with 70% ethanol prior to cell seeding.

For infection, cells were washed twice with PBS and infected with PR8, WSN, WSN-ΔHA:GFP or HK68 virus diluted in FBS-free DMEM/F-12 with an MOI of 3 for 1 h. Then, cells were washed twice with PBS and incubated for 16 h in complete DMEM/F-12. For transfection, a transfection mix was prepared by mixing 200 μl OptiMEM with the respective plasmids (2 μg pCAGGS-HA (PR8) or 0.2 μg pcDNA3.1(-)-mStayGold + 1.8 μg pCAGGS-M1 (HK68) or 0.2 μg pcDNA3.1(-)-mStayGold + 1.8 μg pcDNA3.1( + )-HA (H18N11)) and adding 6 μl TransIT-LT1. The transfection mix was incubated for 20 min at room temperature and then carefully added on top of the cells. Transfected cells were incubated for 24 h before plunge freezing.

Grids were removed from the medium and directly transferred to a Leica GP2 plunge freezer set to 80% chamber humidity and 37 °C chamber temperature. A 3 μl drop of medium from the cell culture dish was added on the top side of the grid. Grids were blotted for 3 s and plunge frozen in liquid ethane at −185 °C.

For CLEM experiments, cells were fixed by exchanging the medium with 4% PFA in PBS and incubating for 15 min at room temperature prior to blotting. Then, the fixative was exchanged with PBS. Prior to blotting, a 3 μl drop of 10% glycerol in PBS was added onto the top side of the grid for 3 min to improve vitrification. Blotting and further steps were done as described above.

### Cryo-FIB milling of infected and transfected cells

Grids were then clipped into ThermoFisher CryoFIB AutoGrids and loaded into an Aquilos 2 cryo-FIB-SEM equipped with an integrated fluorescence light microscope. Target cells were selected in Maps (ThermoFisher Scientific). In case of mStayGold co-transfection, the fluorescence signal was used to target transfected cells. Lamellas were milled with autolamella^61^.

### Correlative light- and electron-microscopy

Cryo-FIB milled lamellae were imaged in a Zeiss LSM 900 Airyscan microscope equipped with a Linkam cryo-stage cooled to −180 °C. First, an overview image was acquired using a 5× air objective in widefield mode. Of each lamella, a stack with 79.5 × 79.5 × 440 nm XYZ pixel spacing was acquired in super-resolution Airyscan mode with a Zeiss EC Epiplan-Neofluar 100×/0.75 DIC objective. Two channel images were acquired, GFP with a 488 nm laser set to 4% intensity and reflection mode using a 640 nm laser set to 0.02% intensity. Stacks were pre-processed using Zen blue (Zeiss) and motion-corrected using ImageJ2 version 2.14.0/1.54 f ^62^. Then, maximum intensity projections were generated in ImageJ2.

Maximum intensity projections were aligned to maps of lamellae in SerialEM prior to tilt series acquisition^63^. For illustration purposes, maps were once more aligned to the same maps after tilt series acquisition using a custom napari^64^ plugin.

### Cryo-electron tomography

Data collection for cryo-ET was done using a Titan Krios Transmission Electron Microscope (TEM, ThermoFisher Scientific) operated at 300 keV and equipped with a BioQuantum® LS energy filter with a slit width of 15 eV and K3 direct electron detector (Gatan) using SerialEM^63^. Montaged maps were acquired at 8700× magnification with pixel size 10.68 Å/px and approximate defocus of −80 μm. If applicable, light microscopy data was aligned to the maps in SerialEM using three registration points. Tilt series were acquired at 33,000× magnification with a pixel size of 2.671 Å/px at defocus between −2.5 and −4 μm defocus, with an electron dose of approximately 3.2 e⁻/Å² per projection. A dose symmetric acquisition scheme was used with a tilt range of +68° to −52° in 3° increments^65^ and tomograms were acquired in parallel with PACE-tomo^66^.

Tomograms were reconstructed with a pixel size of 5.342 Å/px in Etomo^67^. All tomograms were denoised with CryoCare^68^ or IsoNet^69^. Tomogram segmentations were performed in Dragonfly version 2022.1 or 2024.1. Membrane segmentations were performed with MemBrain v2^70,71^ and imported into Dragonfly.

### Subtomogram averaging

A summary of the methods used for subtomogram averaging is shown in Table 1. The subtomogram average of HA was performed in Dynamo^72^. Tomograms were reconstructed using Etomo with a pixel size of 1.68 Å/px. An initial reference was generated by manually picking 96 particles using a dipole model. Then, the bulk of particles was picked using an ellipsoidal vesicle model, oversampling the surface of HA-membranes. Particles were iteratively aligned, while restricting the cone tilt.

**Table 1:** Summary of subtomogram averaging methods used

Protein	Reconstruction	Picking	Refinement	EMDB
HA	Etomo 1.68 Å	Dynamo Initial: dipole model Bulk: ellipsoidal vesicle model	Dynamo	EMD-51790
NA	Etomo 10.684 Å	Dynamo Surface model	Relion 4.0.1	EMD-51741
vRNP	Etomo 10.684 Å	Dynamo Filament with torsion model	Relion 4.0.1	EMD-51742
M1	AreTomo^77^ 10.356 Å	Relion 5.0 beta3 Filament picking in napari^64^	Relion 5.0 beta3	EMD-51740

The subtomogram averages of vRNPs and NA were performed in Dynamo and Relion 4.0.1^73^. Tomograms were reconstructed using Etomo with a pixel size of 10.684 and 5.342 Å/px, respectively, and imported into a Dynamo catalog. vRNPs were picked as filament with torsion models, NA as surface models. After initial refinements in Dynamo, particle tables were transferred to Relion for further refinement and classification. AlphaFold2^33^ was used to predict the structures of NA HK68 and NA PR8. Since the prediction showed an unrealistic arrangement of the NA stem region with transmembrane domain close to the tetrameric domain, the fit in Fig. 1 was performed with an NA model combined from two AlphaFold2 predictions using either only the NA tetrameric head domain or only the stem with transmembrane domain.

The subtomogram average of M1 was performed with Relion 5.0 beta3. Tilt-series movies were imported into Relion, motion-corrected with MotionCor2^74^, CTF-estimated with CTFFind-4.1^75,76^ and aligned with AreTomo^77^. M1 helices were picked using filament picking with a spacing of 110 Å on tomograms reconstructed at 10.356 Å/px.

### Nearest-neighbor clustering analysis

Tomograms were scaled to a pixel-size of 10.684 Å/px and denoised using IsoNet^69^. Segmentations were done in IMOD: membranes were segmented as surface meshes, vRNPs were segmented as open contours. The segmentations were parsed into numpy arrays using the ImodModel package (https://github.com/teamtomo/imodmodel). Pairwise vRNP-vRNP distances were calculated using SciPy version 1.15.3. vRNP-membrane and membrane-membrane distances were calculated using CGAL version 5.6. *P*-values were calculated with SciPy using a 2-sample Kolmogorov-Smirnov test. Nearest neighbor expansion search was performed iteratively in 3D from a randomly selected vRNP using vRNP-vRNP search distance threshold *t* (center-to-center; range 15–26 nm, 18 nm shown in main text). The search is terminated once no neighboring vRNP is found, resulting in a defined cluster. Subsequently, a new randomly selected vRNP is used for clustering analysis until all vRNPs are assigned to clusters. A schematic of the cluster identification algorithm is provided in Supplementary Fig. 3f.

Membrane-membrane distances were measured based on the by calculating the first intersection of the vertex normals with a membrane.

To generate a random vRNP model, the segmented vRNPs were randomly shifted within the tomogram using MTK. All analyses were performed in the same way on the segmented and randomly shifted models.

The data analysis scripts were deposited on GitHub: 10.5281/zenodo.17151506.

### NA sequence analysis

NA sequence and charge analysis was done using ProtPi version 2.2.29.152 (https://www.protpi.ch/Calculator/ProteinTool).

### Sample preparation for quantitative polymerase chain reaction with reverse transcription (RT-qPCR) and Western blot

2 × 10^5^ A549, A549-Rab11wt or A549-Rab11dn cells were seeded in a 24-well plate in either 500 µl Dulbecco’s Modified Eagle Medium (DMEM) 1× supplemented with L-glutamine, 100 U/ml penicillin-streptomycin (P/S), and 10% (v/v) fetal bovine serum (FBS) (for the case of A549 cells) or 500 ml DMEM 1× supplemented with L-glutamine, 1.25 mg/ml puromycin, 100 U/ml penicillin-streptomycin (P/S) and 10% (v/v) fetal bovine serum (FBS).

Cells were incubated for 12 h. Prior to infection, cells were washed with DMEM 1× supplemented with L-glutamine and 100 U/ml penicillin-streptomycin (P/S) before being infected with PR8 virus diluted in FBS-free DMEM with an MOI of 3 for 45 min. Then, cells were washed with acid wash (0.135 M NaCl, 0.01 M KCl, 0.04 M citric acid, pH 3) for 50 s. Cells were then washed with PBS followed by serum free DMEM containing 0.14% bovine serum albumin and incubated at 37 °C and 5% CO_2_.

At each time point of interest, cells were scraped and harvested using 100 µl 2× Laemmli buffer (50% glycerol, 10% SDS, 1 M DTT, 1 M Tris pH 6.8, 2% ethanol containing 2% bromophenol blue and 2% xylene cyanol, H_2_O) for western blot or 300 µl NZYol (NZYtech, MB18501).

### Western blot

At each time point of interest, wells were lysed with 100 µl of 2X Laemmli buffer (see above).

Protein samples were subjected to electrophoresis in either 8% or 12.5% Tris-HCl gels. The resulting bands were transferred on to nitrocellulose membranes (Cytiva, 10600003). Membranes were probed with antibodies directed against PB2 (gift from Jonathan Yewdell, National Institutes of Health, USA), NP (gift from Paul Digard, Roslin Institute, University of Edinburgh, Scotland), HA (gift from Jonathan Yewdell, National Institutes of Health, USA), NS1 (GeneTex, 125990, 1:2000), M1 (Abcam, ab20910, 1:2000), M2 (gift from Jonathan Yewdell, National Institutes of Health, USA) and beta-actin (Merck, A5441, 1:5000) followed by incubation with infrared secondary antibody conjugates with absorbances of 680 nm (LICORBio, 926-68073, 1:10,000) and 800 nm (LICORBio, 926-32210, 1:10000). Images were analyzed using ImageJ2 version 2.14.0/1.54 f.

### Quantitative polymerase chain reaction with reverse transcription (RT-qPCR)

A summary of PCR primers is shown in Table 2. Wells were treated with 300 µl NZYol (NZYtech, MB18501) at the appropriate time points. Cells were then scraped into Eppendorf tubes and stored at −80 °C.

**Table 2:** Summary of primers used for reverse transcription and real-time PCR

Target	Primer (5′-3′)	Purpose
IAV segment 5 vRNA	GGCCGTCATGGTGGCGAAT CTAGCACGGTCTGCACTCAT	Reverse transcription
IAV segment 5 mRNA	CCAGATCGTTCGAGTCGT TTTTTTTTTTTTTTTT TTTAATTGTCG	Reverse transcription
IAV segment 6 vRNA	GGCCGTCATGGTGGCGAAT CTCCGTCCCCGTACAATTCA	Reverse transcription
IAV segment 6 mRNA	CCAGATCGTTCGAGTCGT TTTTTTTTTTTTTTT TGAACAGACTAC	Reverse transcription
vRNA tag	GGCCGTCATGGTGGCGAAT	Real-time PCR
mRNA tag	CCAGATCGTTCGAGTCGT	Real-time PCR
GAPDH	CTCTGCTCCTCCTGTTCGAC	Real-time PCR
GAPDH	ACCAAATCCGTTGACTCCGAC	Real-time PCR
IAV segment 5 vRNA	TCAAAGTCGTACCCACTGGC	Real-time PCR
IAV segment 5 mRNA	CCGATCGTGCCTTCCTTTGA	Real-time PCR
IAV segment 6 vRNA	GATTGTTAGCCAGCCCATGC	Real-time PCR
IAV segment 6 mRNA	TGAATAGTGATACTGTAGATTGGTCT	Real-time PCR

**Table 3:** List of tomograms shown in the main text and EMDB accession codes

Figure	EMDB accession
1a, e, f, g	EMD-50079
1b	EMD-50078
1c	EMD-50067
1d	EMD-50070
1i, j	EMD-50080
1k, l	EMD-51741
1m, n	EMD-51790
1m, n	1RU7
1o, p	4BBL
1o, p	EMD-51742
2a, g	EMD-50083
2b, i	EMD-50082
2c, k	EMD-50081
3c, d, e	EMD-54375
4a, o	EMD-50084
4c, i, k	EMD-50086
4e, m	EMD-50085
4g	EMD-51811
4s, t	EMD-51740
4u–x	6Z5L
4v	EMD-11079
5a, d, e, f, g, h	EMD-50087
5c	EMD-50088

RNA was purified from NZYol treated samples using the Direct-zol RNA miniprep kit (ZYMO, R2052). The reverse transcription reaction was carried out with equal concentrations of RNA per sample using the M-MuLV First-Strand cDNA Synthesis Kit (NZYtech, MB17302). RT-qPCR was implemented in iTaq™ Universal SYBR® Green Supermix (Bio-Rad, 1725121) in a QuantStudio 7 Flex Real-Time PCR System (Thermo Fisher Scientific). GAPDH was used as the reference gene. Analysis was performed using QuantStudio™ Real-Time PCR Software.

**Table 4:** Source data files provided with this paper

Figure	Source data file
Fig. 4m–o Supplementary Fig. 2d–f, h, l	fig_02_mno_sfig_02_defhi_vRNP_nearest_neighbor.csv
Fig. 2p Supplementary Fig. 3f	fig_02_p_03_f_vRNP_cluster_membrane_distances.csv
Fig. 4q	fig_04_q_M1_cylinder_lengths.csv
Fig. 4r	fig_04_r_M1_radial_profile.csv
Fig. 5g	fig_05_g_budding_lengths.csv
Fig. 5r	fig_05_r_WSNdelHA_particle_counts.csv
Fig. 5t	fig_05_t_MDCK_MDCKHA_WSNdelHA_M1-NP-ratio.csv
Fig. 5u	fig_05_u_WSNdelHA_seg5_seg6_vRNA_qpcr.csv
Supplementary Fig. 3a,b,c	sfig_03_abc_hamembrane_quantification.csv
Supplementary Fig. 4b	sfig_04_b_colocalization.csv
Supplementary Fig. 5c	sfig_05_c_PB2_HA_NA_NP_NS1_M1_M2_WB_timeseries.csv
Supplementary Fig. 5d	sfig_05_d_segment_5_6_mRNA_vRNA_qpcr.csv
Supplementary Fig. 6j	sfig_06_j_colocalization.csv

### Reporting summary

Further information on research design is available in the Nature Portfolio Reporting Summary linked to this article.

## Supplementary information

Supplementary Information

Peer Review file

Description of Additional Supplementary Files

Supplementary Movie 1

Supplementary Movie 2

Supplementary Movie 3

Supplementary Movie 4

Supplementary Movie 5

Supplementary Movie 6

Supplementary Movie 7

Reporting Summary

Editorial Policy Checklist - Dynamic

## Source data

Source Data

## Data Availability

All cryo-electron microscopy data generated in this study have been deposited to the EMDB database under the accession codes listed in Table 3. Due to its large size, the confocal microscopy data is available upon request. Source data are provided with this paper (see Table 4 for details). Source data are provided with this paper.

## Code availability

The code to perform the vRNP nearest-neighbor clustering analysis was deposited to GitHub and is available under the 10.5281/zenodo.17151506.
