# Supplementary material for: Visualizing influenza A virus assembly by in situ cryo-electron tomography
Source: Nat Commun. 2025 Oct 23;16:9394. doi: 10.1038/s41467-025-65117-z (PMC12550032; doi:10.1038/s41467-025-65117-z)
Supplement: Supplementary file 1 — Supplementary Information [file 41467_2025_65117_MOESM1_ESM.pdf]

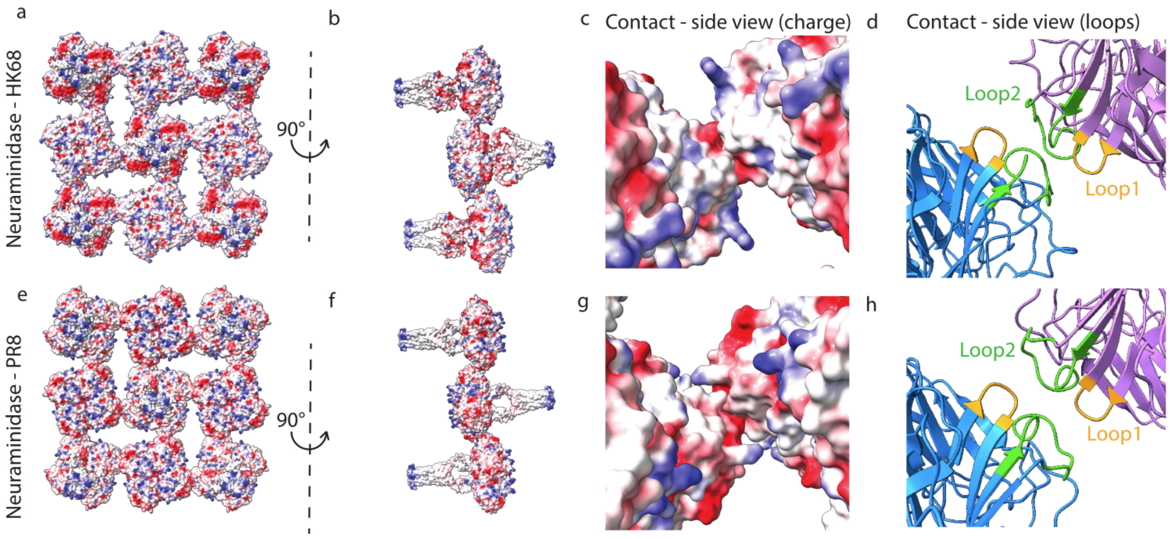

i

NA subtype	Virus	Neuraminidase contacting loop sequence and net charge (pH7)	
		Loop 1	Loop 2
N2	A/Hong Kong/1/68 (H3N2)	N**G**D**D** (-2.6)	**KV**IG**GW**ST**P**N**SK**S (+1.2)
	A/Darwin/9/2021(H3N2)	D**G**N**D** (-1.6)	**KV**VE**GW**SN**P**K**SK**L (+1.1)
	A/Bat/Egypt/381OP/2017(H9N2))	D**G**D**D** (-2.6)	**KV**LQ**GW**VT**P**N**SK**E (+0.15)
N1	A/Puerto Rico/34 (H1N1)	**YGNG** (-0.8)	**WDPNGWTETD**SK**F** (-2.6)
	A//Victoria/4897/2022 (H1N1)	**YGNG** (-0.8)	**WDPNGWTETD**NK**F** (-2.6)
	A/Texas/37/2024 (H5N1)	**YGNG** (-0.8)	**WDPNGWTETD**SS**F** (-3.6)

Supplementary figure 1: Contact sequence analysis of NA-NA zipper. AlphaFold2 predicted structures of HK68-NA **A–D** and PR8-NA **E–H** fitted into the subtomogram average of NA-zippers and visualized in ChimeraX.[77] **C, G** Magnified views of the NA-NA contact depicted as surface with charge (positive charge - blue, negative charge - red, neutral - white). **D, H** Magnified views of the two neighboring NAs (blue and magenta) in the contact site depicted as a ribbon model with loop 1 (green) and loop 2 (yellow). **I** Table detailing NA N1 and N2 subtype viruses and corresponding sequences of loop 1 and loop 2 with a net charge in parentheses. Conserved amino acids are in bold.

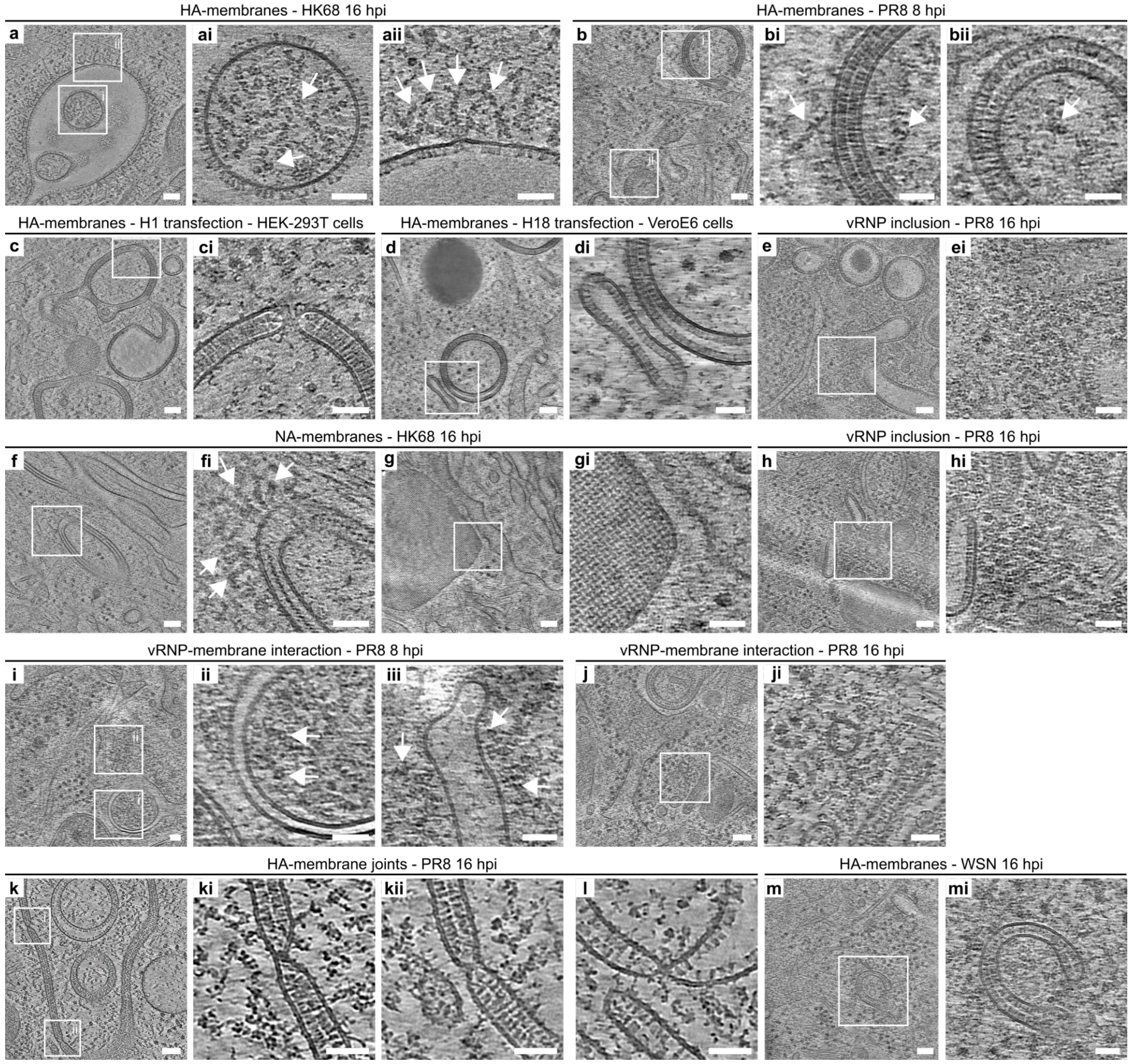

Supplementary figure 2: Cryo-electron tomography slices of HA and NA- membranes in IAV infected or HA transfected cells **A, B** Examples of HA-membranes in a HK68-infected A549wt cell at 16 hpi (A) and a PR8-infected A549wt cell at 8 hpi (B). White arrows indicate vRNPs. **C, D** Example of HA-membranes in a HEK-293T cell ectopically expressing HA (H1) and a VeroE6 cell ectopically expressing H18. **E, H** Examples of viral inclusions composed of vRNP clusters forming a biomolecular condensate and several HA-membranes in a PR8-infected A549wt cell. **F, G** Examples of NA-membranes in a HK68-infected A549wt cell (F) and an A549-Rab11KO cell (G). The top view (G) reveals the C4-symmetric crystalline order. **I** Example of vRNPs (white arrows) interacting with a membrane not containing HA in a PR8-infected A549wt cell at 8 hpi. **J** Example of vRNPs (white arrows) interacting with a vesicle that does not contain HA. **K, L** Examples of constrictions in HA-membranes from PR8-infected

A549-Rab11wt cells. The tomogram shown in (L) is already shown in fig. 2 b. **M** Example of HA-membrane in wild-type WSN virus infected A549 cells. Scale bars: A–L 100 nm; Magnified views 50 nm.

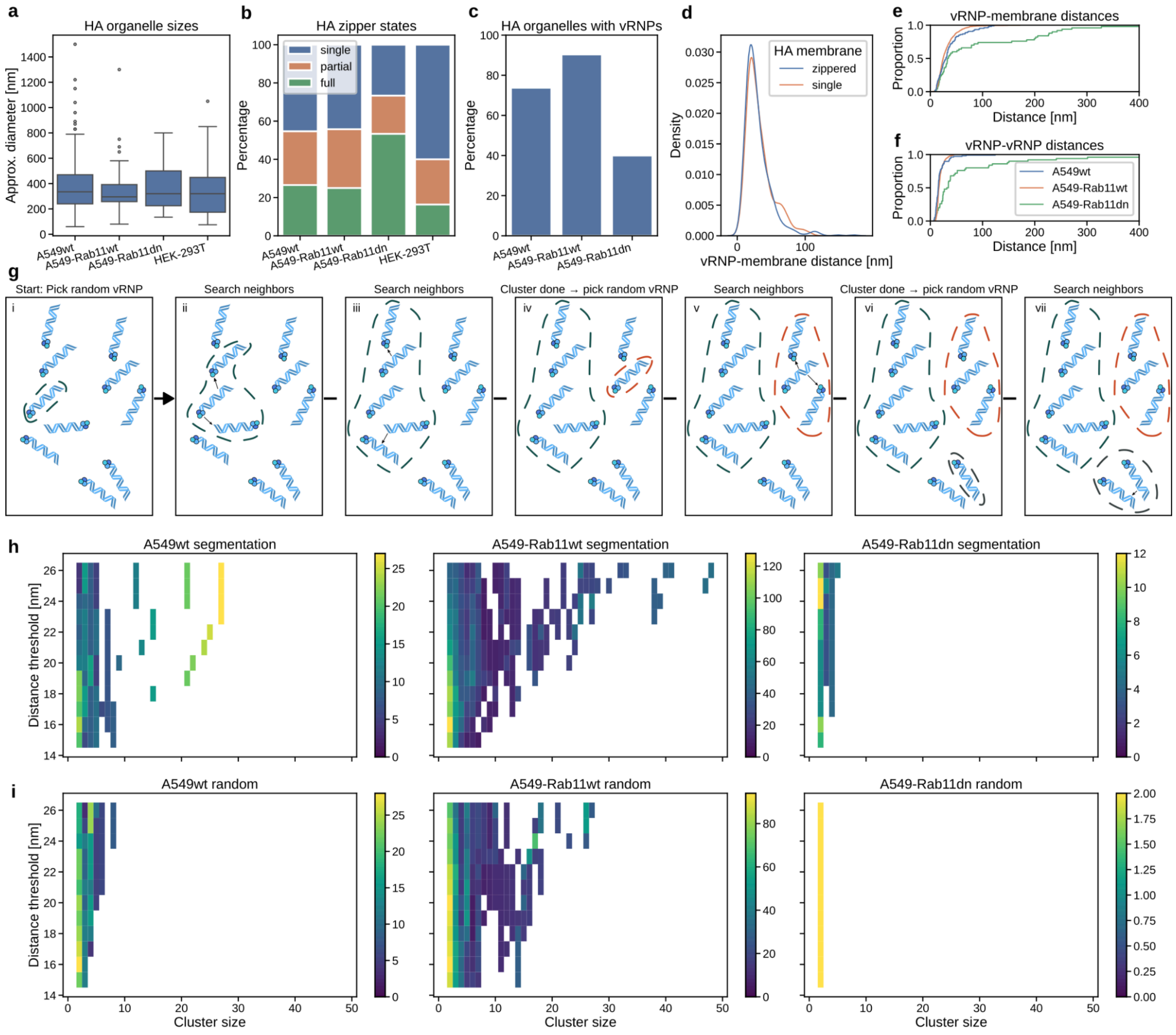

Supplementary figure 3: Quantification of HA-membrane and vRNP spatial analysis. **A** Longest axis lengths of HA-membranes from PR8-infected A549wt (53 tomograms, 128 HA-membranes), A549-Rab11wt (14 tomograms, 52 HA-membranes), A549-Rab11dn (8 tomograms, 15 HA-membranes) and HA-transfected HEK-293T (11 tomograms, 55 HA-membranes) cells. Infected cells at 16 hpi, transfected cells at 24 hpt. Boxes represent the first and third quartiles, whiskers extend to points within 1.5 interquartile ranges. **B** Zipper states of the organelles quantified in (A). Full zippers engage in head-to-head interactions along their entire observable surface, single zippers (HA arrays) do not engage in head-to-head interactions and partial zippers show both phenotypes. **C** Percentage of HA-membranes quantified in (A) with vRNPs

(HA-transfected HEK-293T do not contain vRNPs). **D** Zipper state of the closest membrane patch per vRNP. p=0.15 according to 2-sample Kolmogorov-Smirnov test. **E–F** Shortest distance between vRNP and HA-membranes (D) and between vRNPs (E) for each vRNP (777 vRNPs from 7 tomograms). P-values according to Kolmogorov-Smirnov test: vRNP-vRNP distances 7.7e-4 (A549wt : A549-Rab11wt), 9.5e-10 (A549wt : A549-Rab11dn), 3.0e-13 (A549-Rab11wt : A549-Rab11dn); vRNP-membrane distances 4.5e-2 (A549wt : A549-Rab11wt), 1.6e-2 (A549wt : A549-Rab11dn), 9.9e-5 (A549-Rab11wt : A549-Rab11dn) **G** Schematic of the iterative cluster identification algorithm. The iterative process is performed as follows: (i) Cluster defined by a randomly selected vRNP expanded with all vRNPs closer than the distance threshold t (ii). This is repeated until no more vRNPs are found that are closer than t, at which point the cluster is completed (iii). This is repeated until all vRNPs are assigned to a cluster (iv–vii). For illustration, each vRNP cluster with 3 or more vRNPs is assigned a unique color, clusters of 1 or 2 vRNPs are colored gray. **H** vRNP cluster sizes are compared between A549wt, A549-Rab11wt and A549-Rab11dn cells and between different center-to-center distance thresholds. Single vRNPs (cluster size = 1) are not shown. The dashed line indicates a cluster size of 8 vRNPs. **I** vRNPs quantified in (F) were randomly shifted and subjected to the same quantification. All data plotted with seaborn version 0.13.2. Source data are provided as a Source Data file.

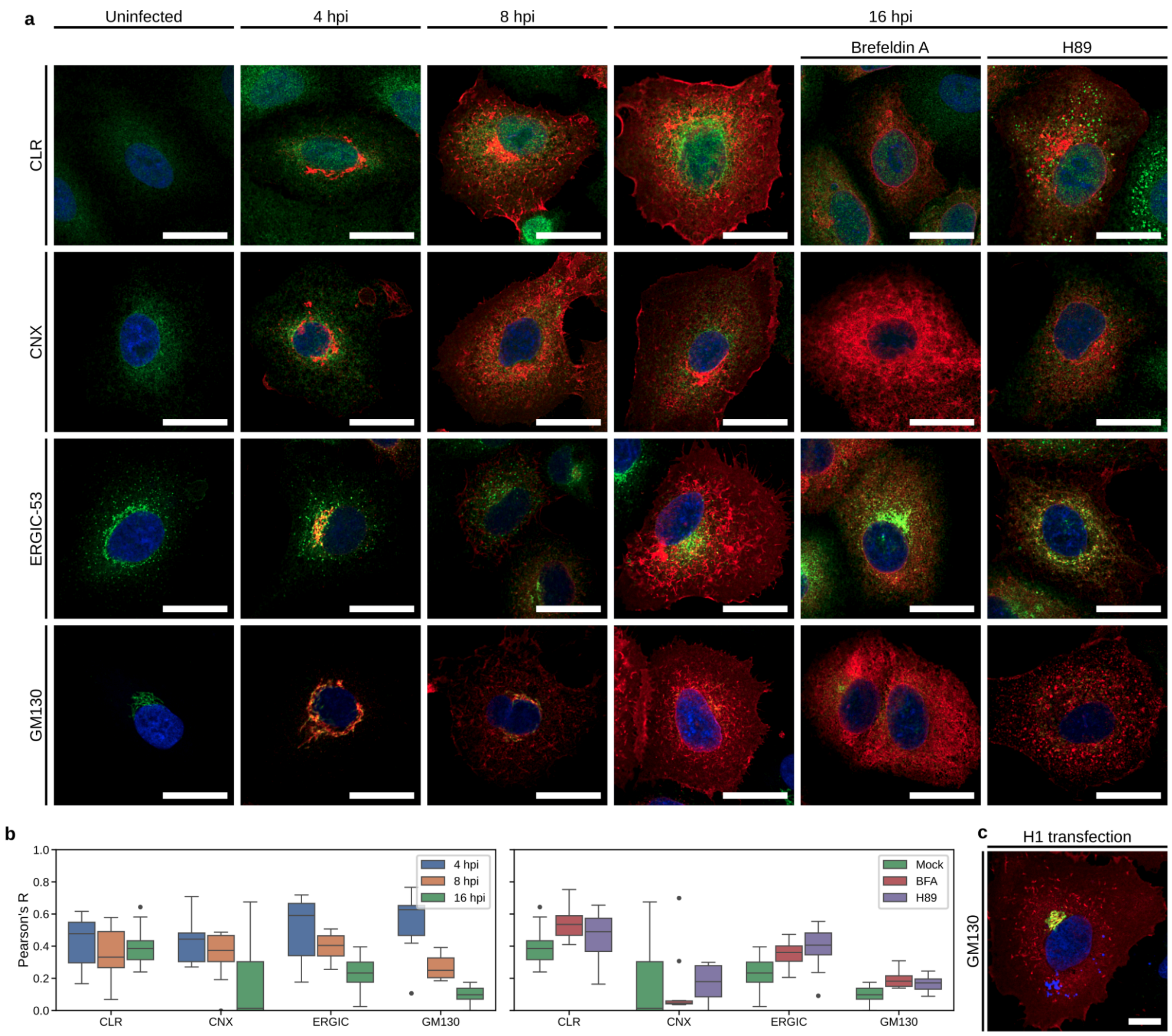

Supplementary figure 4: Confocal microscopy and colocalization analysis of HA-membranes with ER, ERGIC and Golgi apparatus markers. A Representative immunofluorescence confocal microscopy slices of PR8-infected and mock, brefeldin A or H89-treated A549wt cells stained against HA (red) and either calreticulin (CLR) or calnexin (CNX) (ER markers), ERGIC-53 (ERGIC marker) or GM130 (Golgi marker) (green). Nucleus stained with DAPI is shown in blue. B Pearson's correlation coefficients of HA with each membrane marker shown in A, ten cells analyzed per condition. Boxes represent the first and third quartiles, whiskers extend to points within 1.5 interquartile ranges. C A549wt cell transfected with PR8 H1 stained against HA (red) and GM130 (green) at 24 hpt. All data plotted with seaborn version 0.13.2. Source data are provided as a Source Data file. Scale bars: 20 µm

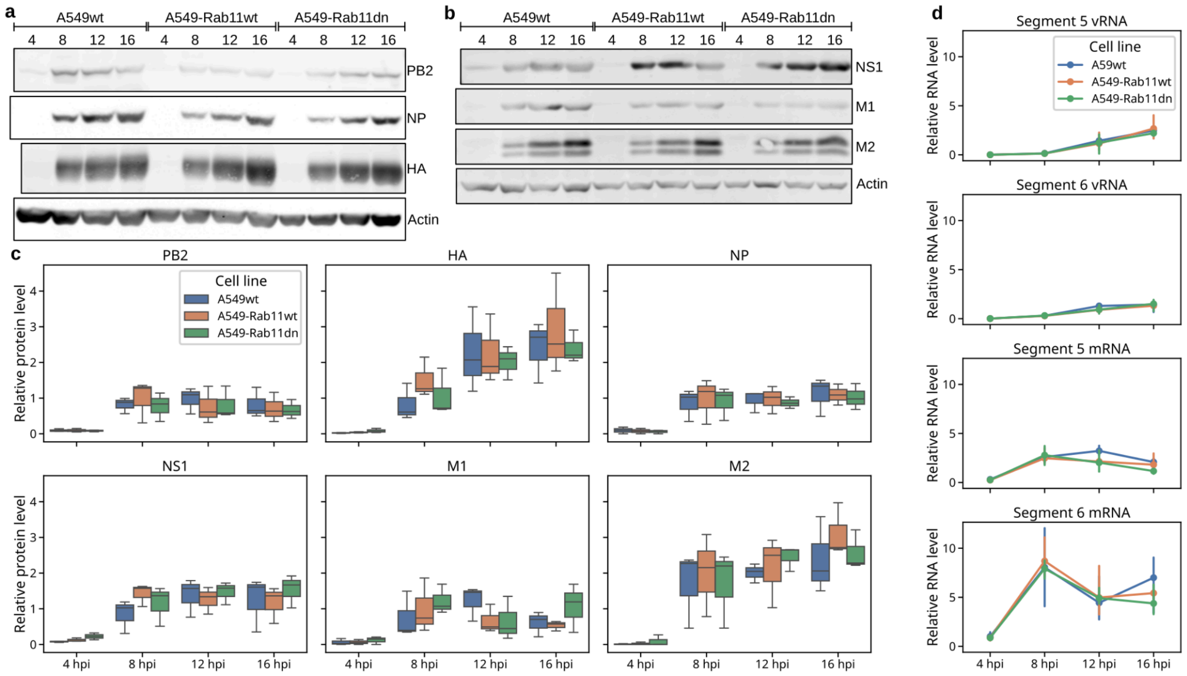

Supplementary figure 5: Viral proteins, viral RNA and mRNA levels during the course of infection of A549wt, A549-Rab11wt, and A549-Rab11dn cells. **A, B** Representative western blots using antibodies against PB2, HA, NP, NS1, M1, and M2 proteins and actin of lysates obtained from PR8-infected A549wt, A549-Rab11wt and A549-Rab11dn cells at different times post-infection. **C** Average viral protein levels normalized to actin quantified from western blots (3 independent replicates, representative images shown in A and B) during the course of infection in A549wt, A549-Rab11wt and A549-Rab11dn cells infected with PR8. Boxes represent the first and third quartiles, whiskers extend to points within 1.5 interquartile ranges. **D** mRNA and vRNA (segments 5 and 6) levels normalized to GAPDH mRNA quantified by qPCR in A549wt, A549-Rab11wt and A549-Rab11dn cells infected with PR8 at 4, 8, 12 and 16 hpi. Data from three independent experiments, error bars indicate 95% confidence intervals. All data plotted with seaborn version 0.13.2. Source data are provided as a Source Data file.

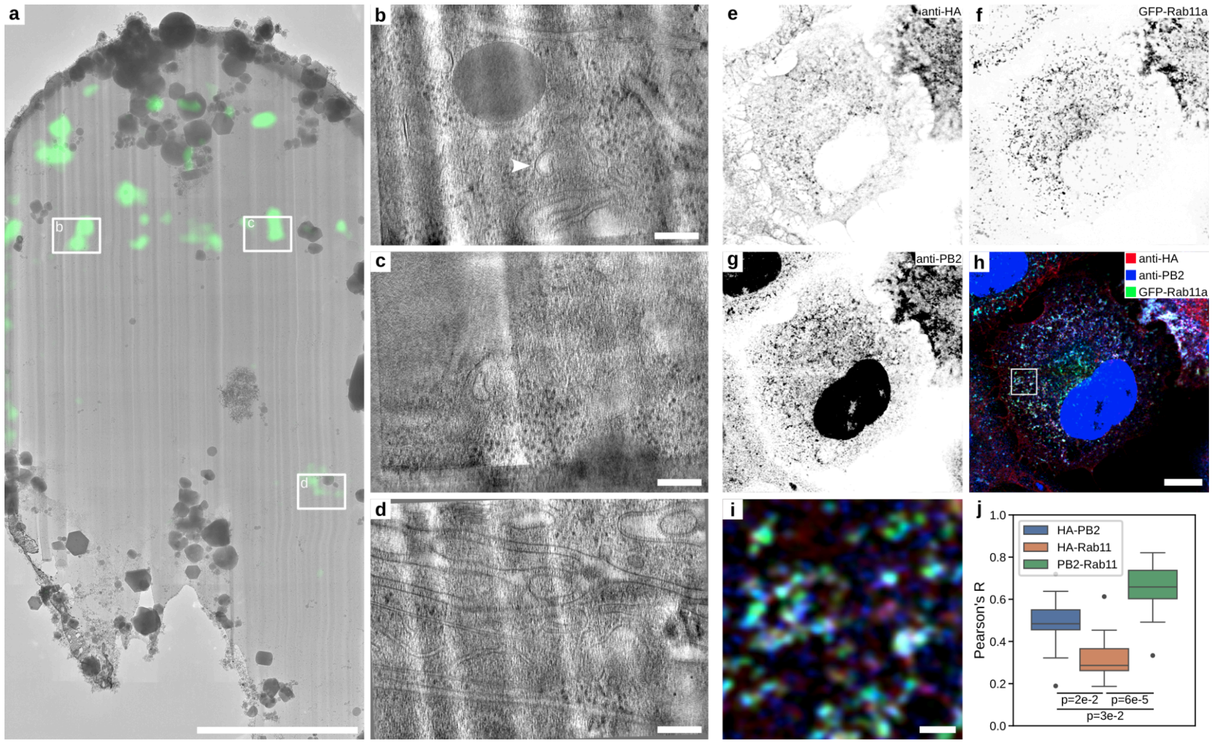

Supplementary figure 6: Cryo-correlative light and electron microscopy and colocalization analysis of HA-positive vesicles with Rab11a and PB2 in A549 cells. A Overview map of a cryo-lamella of a PR8-infected A549-Rab11wt cell overlaid with GFP fluorescence signal. B–D Slices through tomograms acquired at the indicated areas shown in (A). HA-membranes are indicated by black arrowheads. Scale bars: A 5 µm; B–D 100 nm. E–I A549-Rab11wt cells were infected with PR8 (MOI = 5), fixed at 16 hpi and processed for immunofluorescence staining of HA and PB2 (as proxy for vRNPs). J Pearson's colocalization analysis of HA, PB2 and Rab11, restricted to the cytoplasm. Boxes represent the first and third quartiles, whiskers extend to points within 1.5 interquartile ranges. 10 cells were analyzed, p-values are determined by a two-sided T-test. Scale bars: H 10 µm; I 1 µm. All data plotted with seaborn version 0.13.2. Source data are provided as a Source Data file.

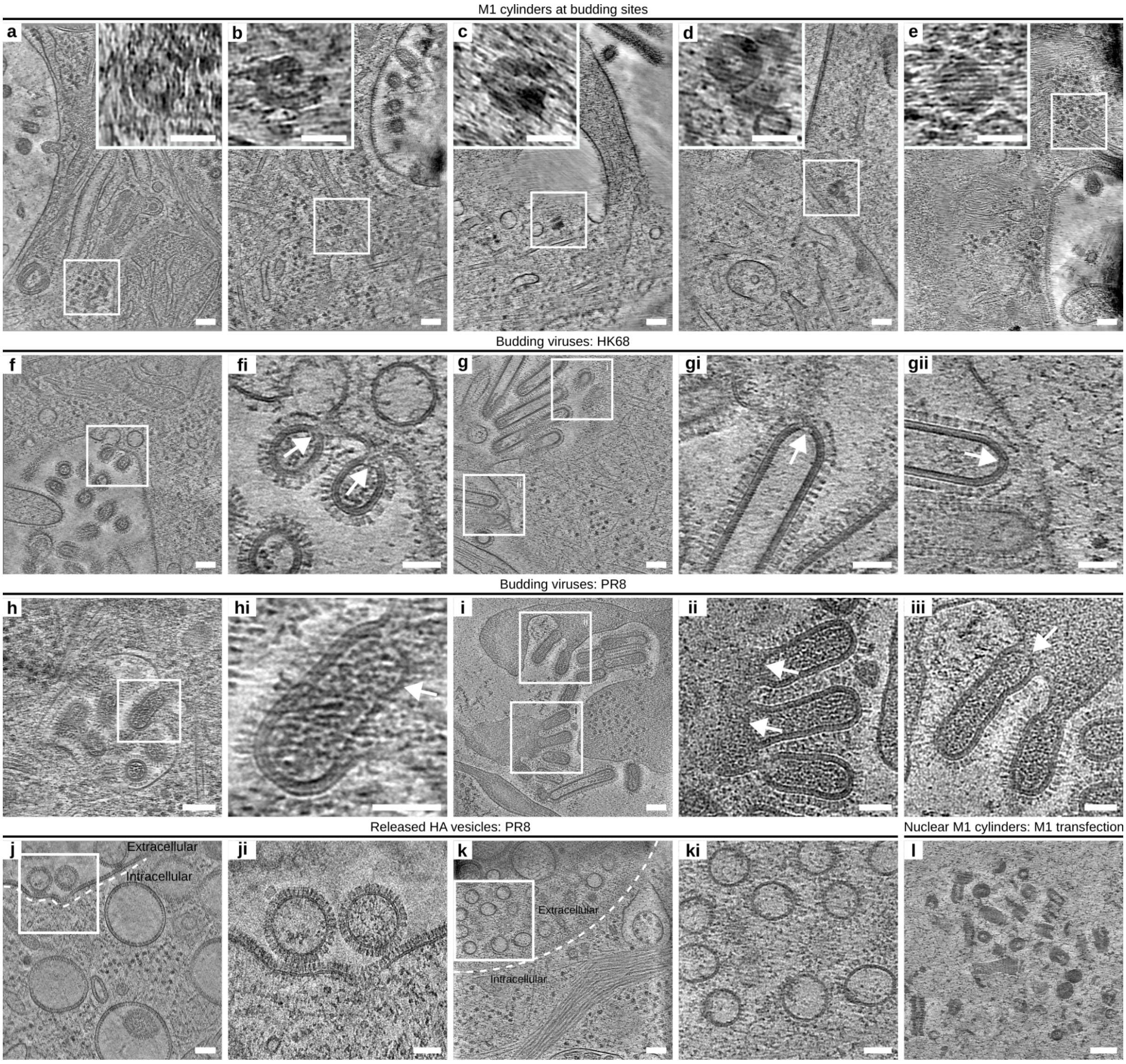

Supplementary figure 7: Cryo-ET of M1 cylinders and budding PR8 and HK68 virions. **A–E** Slices through cryo-electron tomograms showing M1 cylinders close to virus budding sites (B, C, E) or plasma membranes (A, D). **F, G** Slices of tomograms showing budding of spherical (F) and filamentous (G) HK68 virions. Gaps in the M1 layer at the trailing end of the virion are indicated by a white arrow. **H, I** Slices of tomograms obtained by cryo-ET of cryo-FIB milled cells (H) or whole cell cryo-ET (J) of PR8 budding virions forming short-filamentous virions. M1 layers that are not attached to the membrane of the budding virion are indicated by white arrows. **J, K** Slices of tomograms showing release HA-containing vesicles at the extracellular area of an infected cell. **L** Slice of a tomogram of a M1 (HK68) transfected VeroE6 cell showing M1 cylinders in the nucleus. Scale bars: 100 nm; Magnified views 50 nm.
