# Supplementary material for: Visualizing influenza A virus assembly by in situ cryo-electron tomography
Source: Nat Commun. 2025 Oct 23;16:9394. doi: 10.1038/s41467-025-65117-z (PMC12550032; doi:10.1038/s41467-025-65117-z)
Supplement: Supplementary file 3 — Description of Additional Supplementary Files [file 41467_2025_65117_MOESM3_ESM.pdf]

File Name: Supplementary Movie 1
Description: Slices through a cryo-electron tomogram of an A549 cell infected with HK68 at 16 hours post-infection showing a NA-zipper.

File Name: Supplementary Movie 2
Description: Slices through a cryo-electron tomogram and manual segmentation of an A549 cell infected with PR8 at 16 hours post-infection showing an HA-zipper and vRNPs.

File Name: Supplementary Movie 3
Description: Subtomogram average of zippered influenza A virus neuraminidase.

File Name: Supplementary Movie 4
Description: Slices through a cryo-electron tomogram of an A549 cell infected with PR8 showing vRNPs associated with HA-membranes or membranes lacking HA, forming a zone devoid of any other cellular material, resembling a biomolecular condensate.

File Name: Supplementary Movie 5
Description: Slices through a cryo-electron tomogram of an A549 cell infected with PR8 showing vRNPs associated with HA-membranes or membranes lacking HA, forming a zone devoid of any other cellular material, resembling a biomolecular condensate.

File Name: Supplementary Movie 6
Description: Slices through a cryo-electron tomogram of an A549 cell infected with PR8 at 8 hours post-infection showing M1 helices in the nucleus with vRNPs in the vicinity.

File Name: Supplementary Movie 7
Description: Slices through a cryo-electron tomogram of an A549 cell infected with PR8 at 16 hours post-infection showing loosely coiled M1 helices in the cytoplasm.
