# Supplementary material for: Visualizing influenza A virus assembly by in situ cryo-electron tomography
Source: Nat Commun. 2025 Oct 23;16:9394. doi: 10.1038/s41467-025-65117-z (PMC12550032; doi:10.1038/s41467-025-65117-z)
Supplement: Supplementary file 11 — Reporting Summary [file 41467_2025_65117_MOESM11_ESM.pdf]

nature portfolio

Corresponding author(s): Petr Chlanda

Last updated by author(s): 09/19/2025

Reporting Summary

Nature Portfolio wishes to improve the reproducibility of the work that we publish. This form provides structure for consistency and transparency in reporting. For further information on Nature Portfolio policies, see our Editorial Policies and the Editorial Policy Checklist.

Statistics

For all statistical analyses, confirm that the following items are present in the figure legend, table legend, main text, or Methods section.

n/a	Confirmed	
☐	☒	The exact sample size (*n*) for each experimental group/condition, given as a discrete number and unit of measurement
☐	☒	A statement on whether measurements were taken from distinct samples or whether the same sample was measured repeatedly
☐	☒	The statistical test(s) used AND whether they are one- or two-sided<br>*Only common tests should be described solely by name; describe more complex techniques in the Methods section.*
☒	☐	A description of all covariates tested
☒	☐	A description of any assumptions or corrections, such as tests of normality and adjustment for multiple comparisons
☐	☒	A full description of the statistical parameters including central tendency (e.g. means) or other basic estimates (e.g. regression coefficient) AND variation (e.g. standard deviation) or associated estimates of uncertainty (e.g. confidence intervals)
☐	☒	For null hypothesis testing, the test statistic (e.g. *F*, *t*, *r*) with confidence intervals, effect sizes, degrees of freedom and *P* value noted<br>*Give P values as exact values whenever suitable.*
☒	☐	For Bayesian analysis, information on the choice of priors and Markov chain Monte Carlo settings
☒	☐	For hierarchical and complex designs, identification of the appropriate level for tests and full reporting of outcomes
☐	☒	Estimates of effect sizes (e.g. Cohen's *d*, Pearson's *r*), indicating how they were calculated

Our web collection on statistics for biologists contains articles on many of the points above.

Software and code

Policy information about availability of computer code

Data collection	SerialEM 4.0.1<br>Zeiss ZenBlue<br>Leica LAS X
Data analysis	MTK (imod 4.12.56)<br>https://github.com/Chlanda-Lab/nearest_neighbor_cluster_analysis

For manuscripts utilizing custom algorithms or software that are central to the research but not yet described in published literature, software must be made available to editors and reviewers. We strongly encourage code deposition in a community repository (e.g. GitHub). See the Nature Portfolio guidelines for submitting code & software for further information.

Data

Policy information about availability of data

All manuscripts must include a data availability statement. This statement should provide the following information, where applicable:

- Accession codes, unique identifiers, or web links for publicly available datasets
- A description of any restrictions on data availability
- For clinical datasets or third party data, please ensure that the statement adheres to our policy

All cryo-electron microscopy data generated in this study have been deposited to the EMDB database under the accession codes listed in table 4. Due to the large size, confocal microscopy data are available upon request.

Research involving human participants, their data, or biological material

Policy information about studies with human participants or human data. See also policy information about sex, gender (identity/presentation), and sexual orientation and race, ethnicity and racism.

Reporting on sex and gender	This data has not been collected
Reporting on race, ethnicity, or other socially relevant groupings	This data has not been collected
Population characteristics	This data has not been collected
Recruitment	This data has not been collected
Ethics oversight	No ethics oversight

Note that full information on the approval of the study protocol must also be provided in the manuscript.

Field-specific reporting

Please select the one below that is the best fit for your research. If you are not sure, read the appropriate sections before making your selection.

☒ Life sciences ☐ Behavioural & social sciences ☐ Ecological, evolutionary & environmental sciences

For a reference copy of the document with all sections, see nature.com/documents/nr-reporting-summary-flat.pdf

Life sciences study design

All studies must disclose on these points even when the disclosure is negative.

Sample size	HA-remodelled membrane classification/size measurements: all available data, 86 tomograms<br>vRNP nearest-neighbor analysis: 1-3 samples per cell line, 777 analyzed vRNPs in total<br>Correlative light and electron microscopy: 1 experiment, 7 samples<br>Immunofluorescence microscopy: 1 biological replicate and 10 cells analyzed per condition
Data exclusions	Tomograms that were too noisy which prevented unambiguous analysis were excluded.
Replication	Infection experiments for cryo-ET were performed at 8 and 16 hour post infection and using two different viruses and different cell lines. Samples were prepared in multiple technical replications (n=3-5 EM grids) and as 2 independent biological replicates.
Randomization	No randomization performed
Blinding	Blinding was not performed, however, the data were examined independetelly by two persons

Reporting for specific materials, systems and methods

We require information from authors about some types of materials, experimental systems and methods used in many studies. Here, indicate whether each material, system or method listed is relevant to your study. If you are not sure if a list item applies to your research, read the appropriate section before selecting a response.

Materials & experimental systems

n/a	Involved in the study
	[x] Antibodies
	[x] Eukaryotic cell lines
[x]	Palaeontology and archaeology
[x]	Animals and other organisms
[x]	Clinical data
[x]	Dual use research of concern
[x]	Plants

Methods

n/a	Involved in the study
[x]	ChIP-seq
[x]	Flow cytometry
[x]	MRI-based neuroimaging

Antibodies

Antibodies used

mouse anti-HA stalk-binding antibody MEDI8852 1:500
Genetex rabbit anti-PB2 GTX125926 lot no. 44902 1:1000
Cell Signaling rabbit anti-Rab11a 2413S 1:50
StressMarq rabbit anti-calreticulin SPC-122 1:100
Cell Signaling rabbit anti-calnexin 2679S 1:100
Proteintech rabbit anti-ERGIC-53 13364-1-AP lot no. 00114367 1:500
Cell Signaling rabbit anti-GM130 12480S lot no. 4 1:100

Invitrogen A11034 Alexa Fluor 488 goat anti-rabbit lot no. 2110499 1:1000
Invitrogen A11030 Alexa Fluor 546 goat anti-mouse lot no. 2026145 1:1000
Invitrogen A11010 Alexa Fluor 546 goat anti-rabbit lot no. 2189179 1:1000
Invitrogen A21052 Alexa Fluor 633 goat anti-mouse lot no. 2126815 1:1000

Validation

Describe the validation of each primary antibody for the species and application, noting any validation statements on the manufacturer's website, relevant citations, antibody profiles in online databases, or data provided in the manuscript.

Eukaryotic cell lines

Policy information about cell lines and Sex and Gender in Research

Cell line source(s)

A549wt and HEK-293T: ATCC
A549-Rab11wt, A549-Rab11dn, MDCK: Prof. Maria João Amorim, Instituto Gulbenkian de Ciência, Portugal
A549-Rab11KO: Prof. Balaji Manicassami, University of Iowa, USA
Vero E6 from ATCC CRL-1586

Authentication

Cell lines were not authenticated but A549 cells were purchased from ATCC which guarantee their authenticity

Mycoplasma contamination

All cell lines were tested negative for mycoplasma contamination

Commonly misidentified lines
(See ICLAC register)

HEK: use solely in validation experiment due to high transfection efficiency; no HeLa cells used in the lab

Plants

Seed stocks

Report on the source of all seed stocks or other plant material used. If applicable, state the seed stock centre and catalogue number. If plant specimens were collected from the field, describe the collection location, date and sampling procedures.

Novel plant genotypes

Describe the methods by which all novel plant genotypes were produced. This includes those generated by transgenic approaches, gene editing, chemical/radiation-based mutagenesis and hybridization. For transgenic lines, describe the transformation method, the number of independent lines analyzed and the generation upon which experiments were performed. For gene-edited lines, describe the editor used, the endogenous sequence targeted for editing, the targeting guide RNA sequence (if applicable) and how the editor was applied.

Authentication

Describe any authentication procedures for each seed stock used or novel genotype generated. Describe any experiments used to assess the effect of a mutation and, where applicable, how potential secondary effects (e.g. second site T-DNA insertions, mosiacism, off-target gene editing) were examined.
