# Supplementary material for: Visualizing influenza A virus assembly by in situ cryo-electron tomography
Source: Nat Commun. 2025 Oct 23;16:9394. doi: 10.1038/s41467-025-65117-z (PMC12550032; doi:10.1038/s41467-025-65117-z)
Supplement: Supplementary file 13 — Source Data [file 41467_2025_65117_MOESM13_ESM.zip › western_blots_uncropped/WB_summary.pptx]

## Slide 1
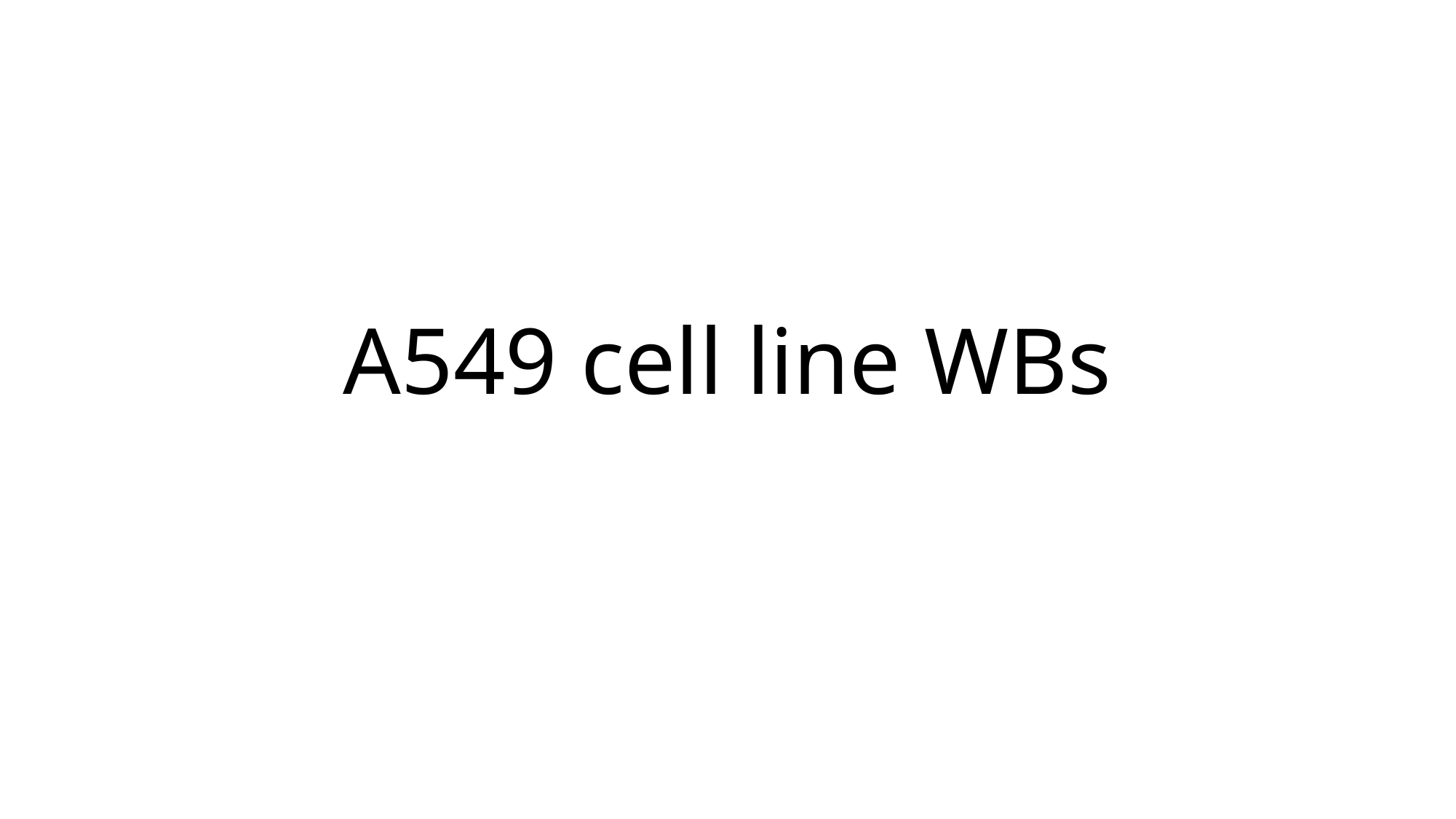

# A549 cell line WBs

## Slide 2
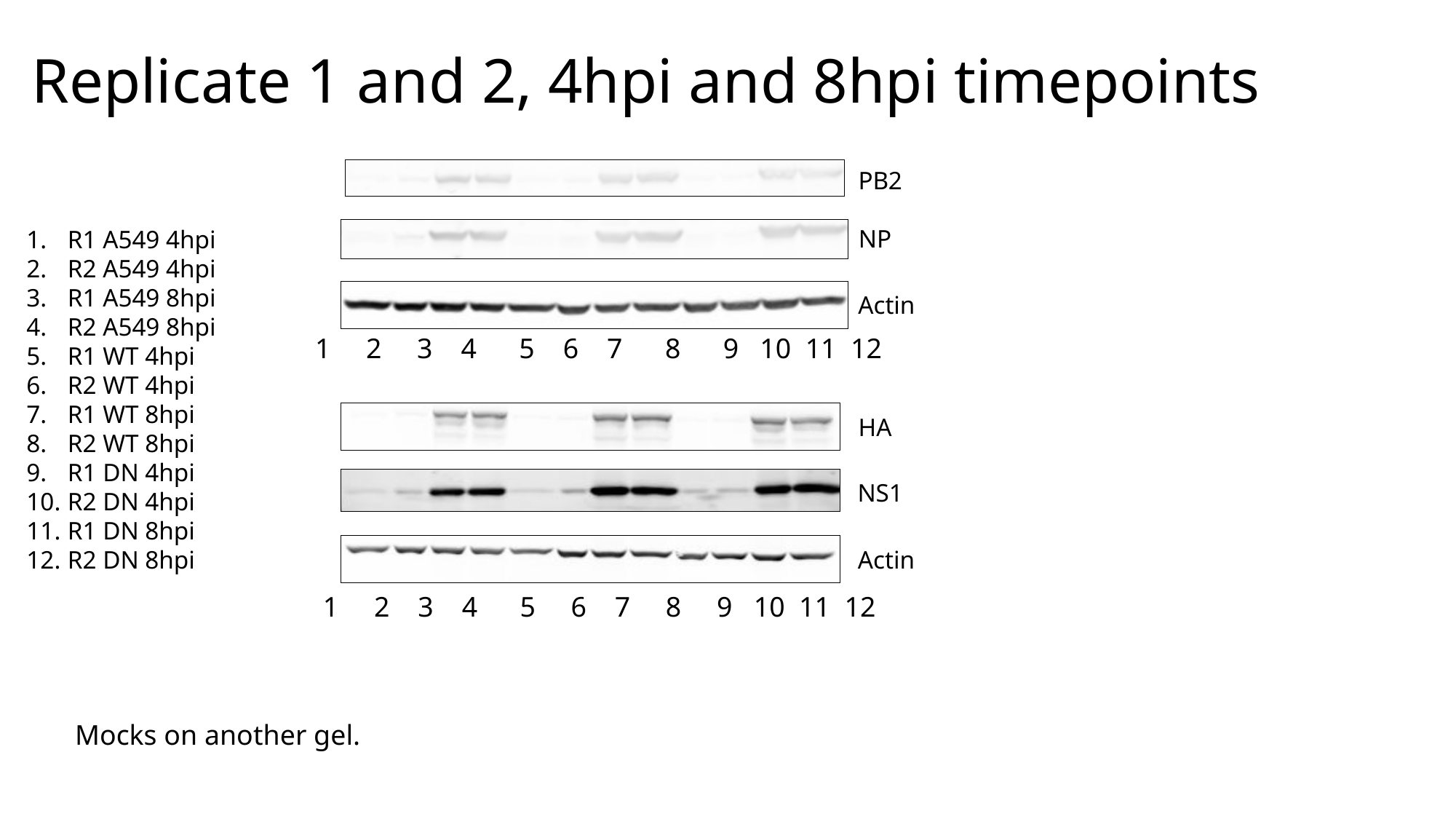

# Replicate 1 and 2, 4hpi and 8hpi timepoints
PB2
NP
R1 A549 4hpi
R2 A549 4hpi
R1 A549 8hpi
R2 A549 8hpi
R1 WT 4hpi
R2 WT 4hpi
R1 WT 8hpi
R2 WT 8hpi
R1 DN 4hpi
R2 DN 4hpi
R1 DN 8hpi
R2 DN 8hpi
Actin
 1 2 3 4 5 6 7 8 9 10 11 12
HA
NS1
Actin
 1 2 3 4 5 6 7 8 9 10 11 12
Mocks on another gel.

## Slide 3
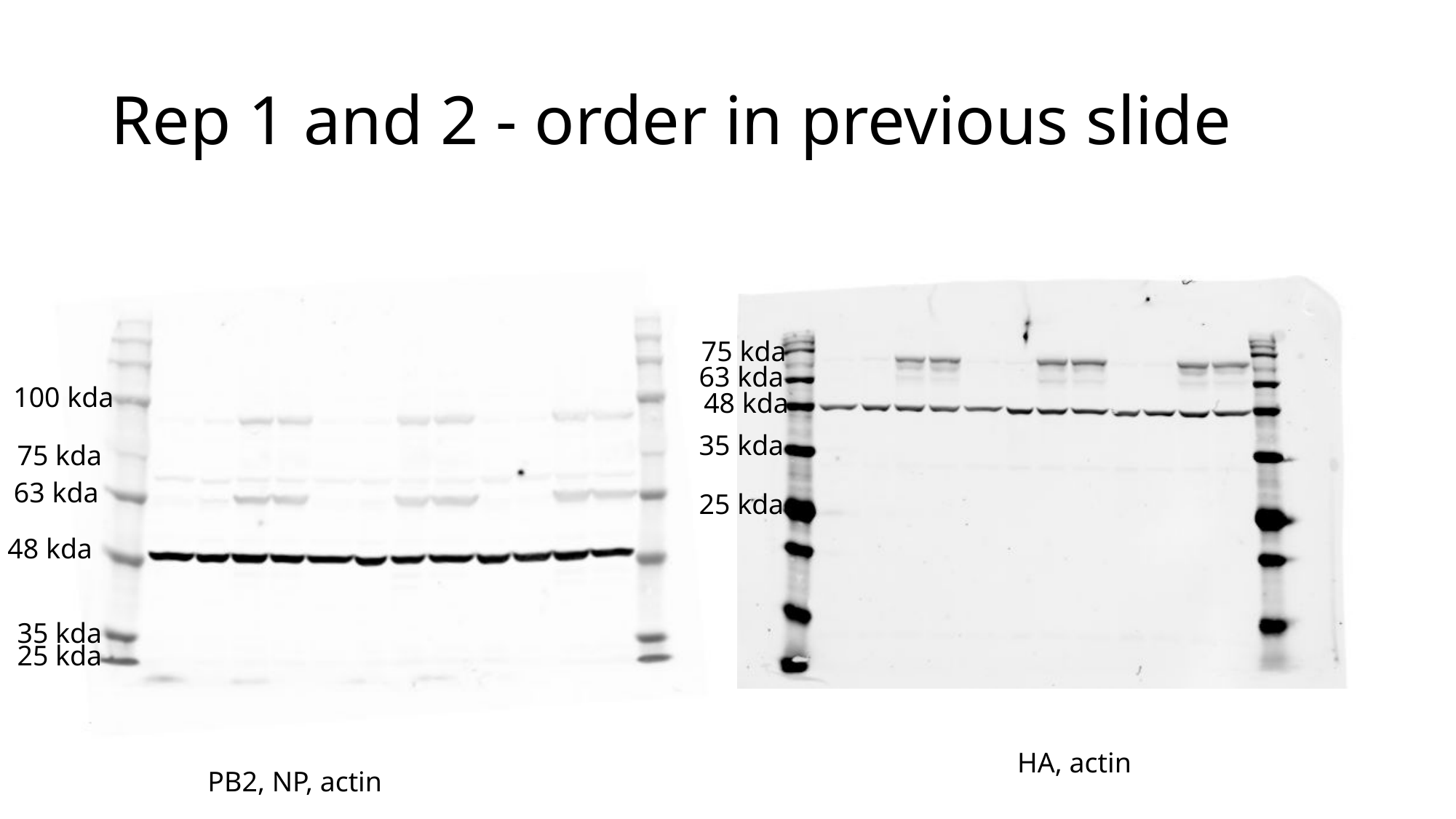

# Rep 1 and 2 - order in previous slide
75 kda
63 kda
100 kda
48 kda
35 kda
75 kda
63 kda
25 kda
48 kda
35 kda
25 kda
HA, actin
PB2, NP, actin

## Slide 4
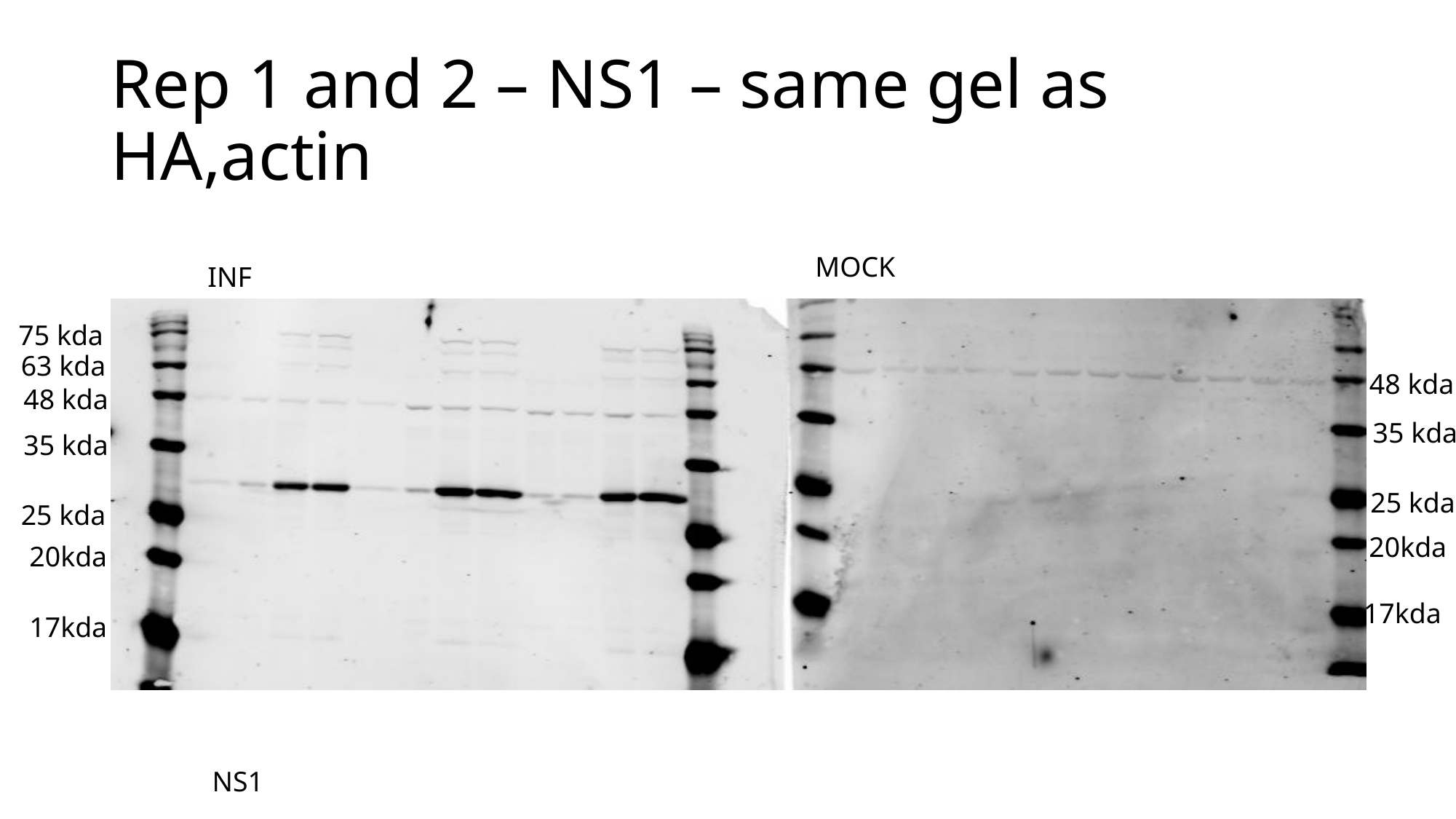

# Rep 1 and 2 – NS1 – same gel as HA,actin
MOCK
INF
75 kda
63 kda
48 kda
48 kda
35 kda
35 kda
25 kda
25 kda
20kda
20kda
17kda
17kda
NS1

## Slide 5
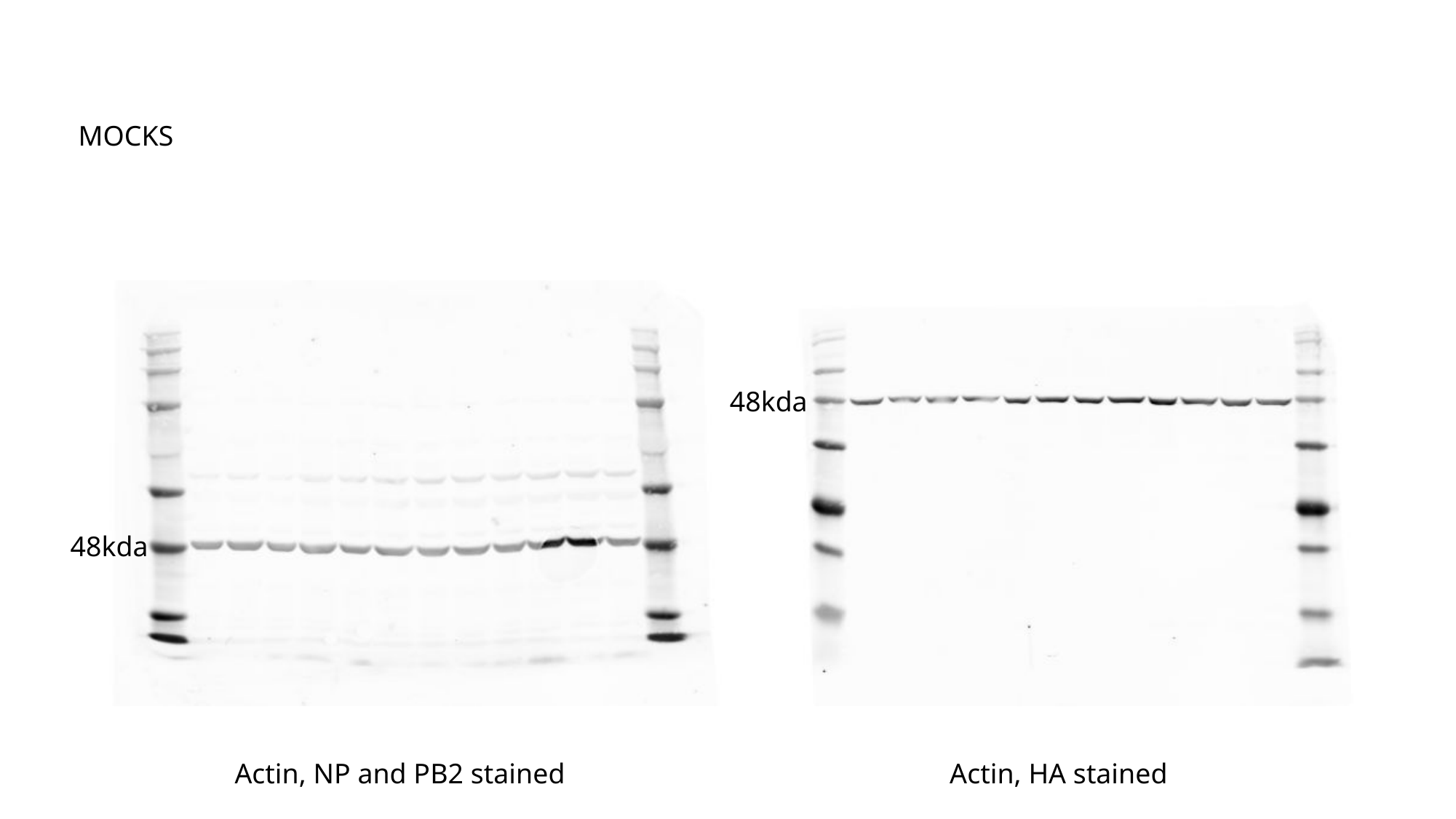

MOCKS
48kda
48kda
Actin, NP and PB2 stained
Actin, HA stained

## Slide 6
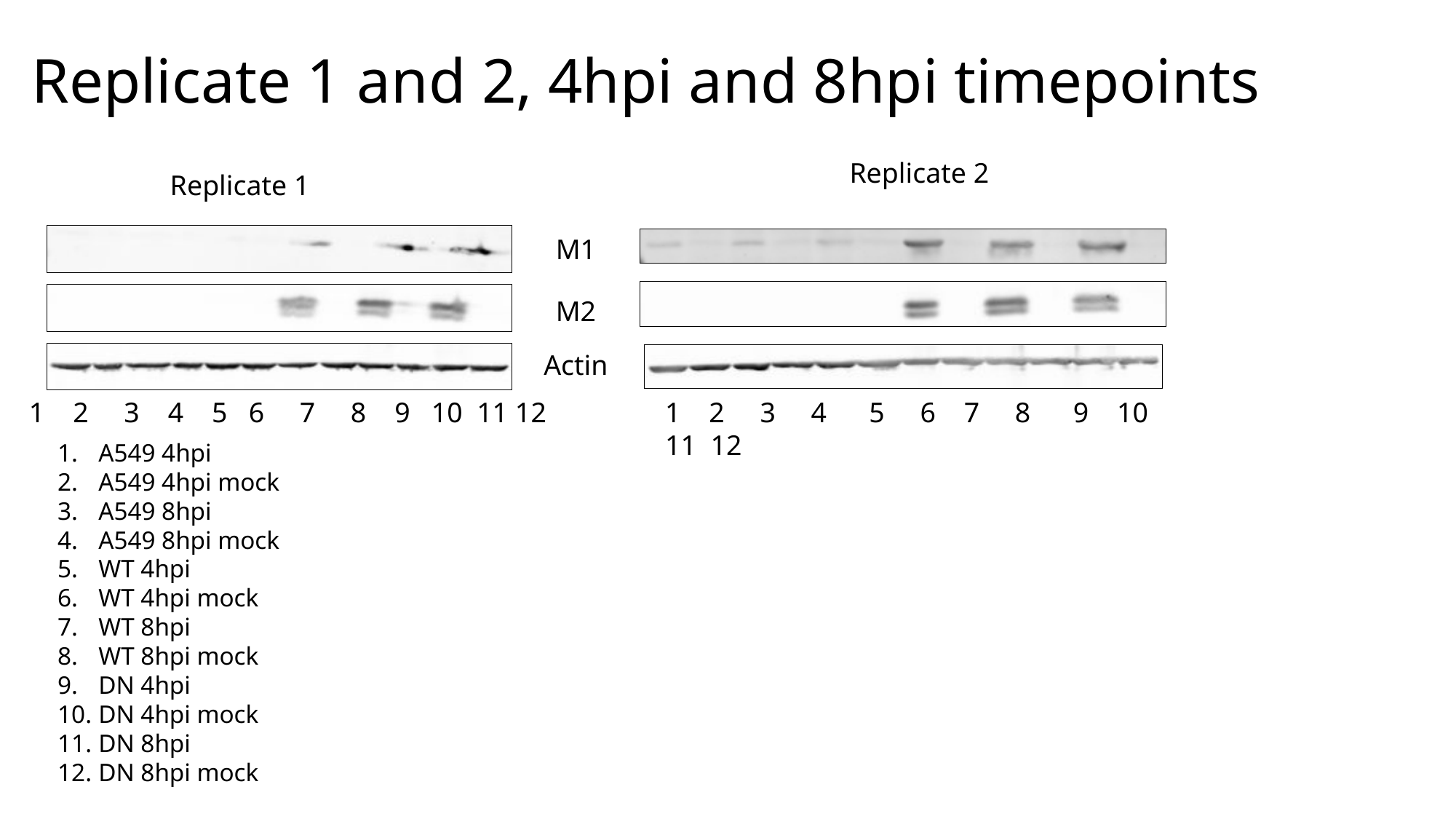

# Replicate 1 and 2, 4hpi and 8hpi timepoints
Replicate 2
Replicate 1
M1
M2
Actin
 1 2 3 4 5 6 7 8 9 10 11 12
 1 2 3 4 5 6 7 8 9 10 11 12
A549 4hpi
A549 4hpi mock
A549 8hpi
A549 8hpi mock
WT 4hpi
WT 4hpi mock
WT 8hpi
WT 8hpi mock
DN 4hpi
DN 4hpi mock
DN 8hpi
DN 8hpi mock

## Slide 7
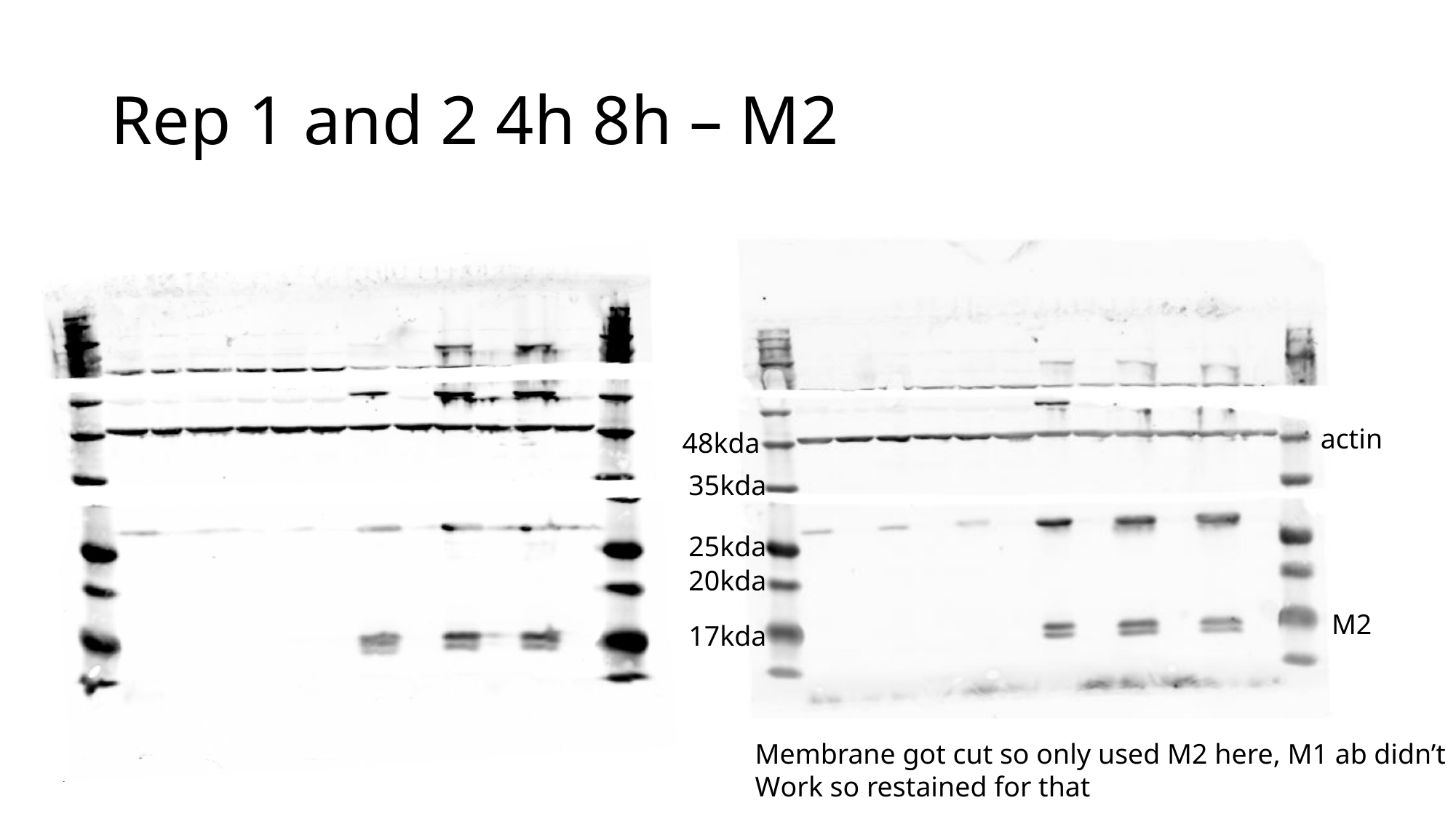

# Rep 1 and 2 4h 8h – M2
actin
48kda
35kda
25kda
20kda
M2
17kda
Membrane got cut so only used M2 here, M1 ab didn’t
Work so restained for that

## Slide 8
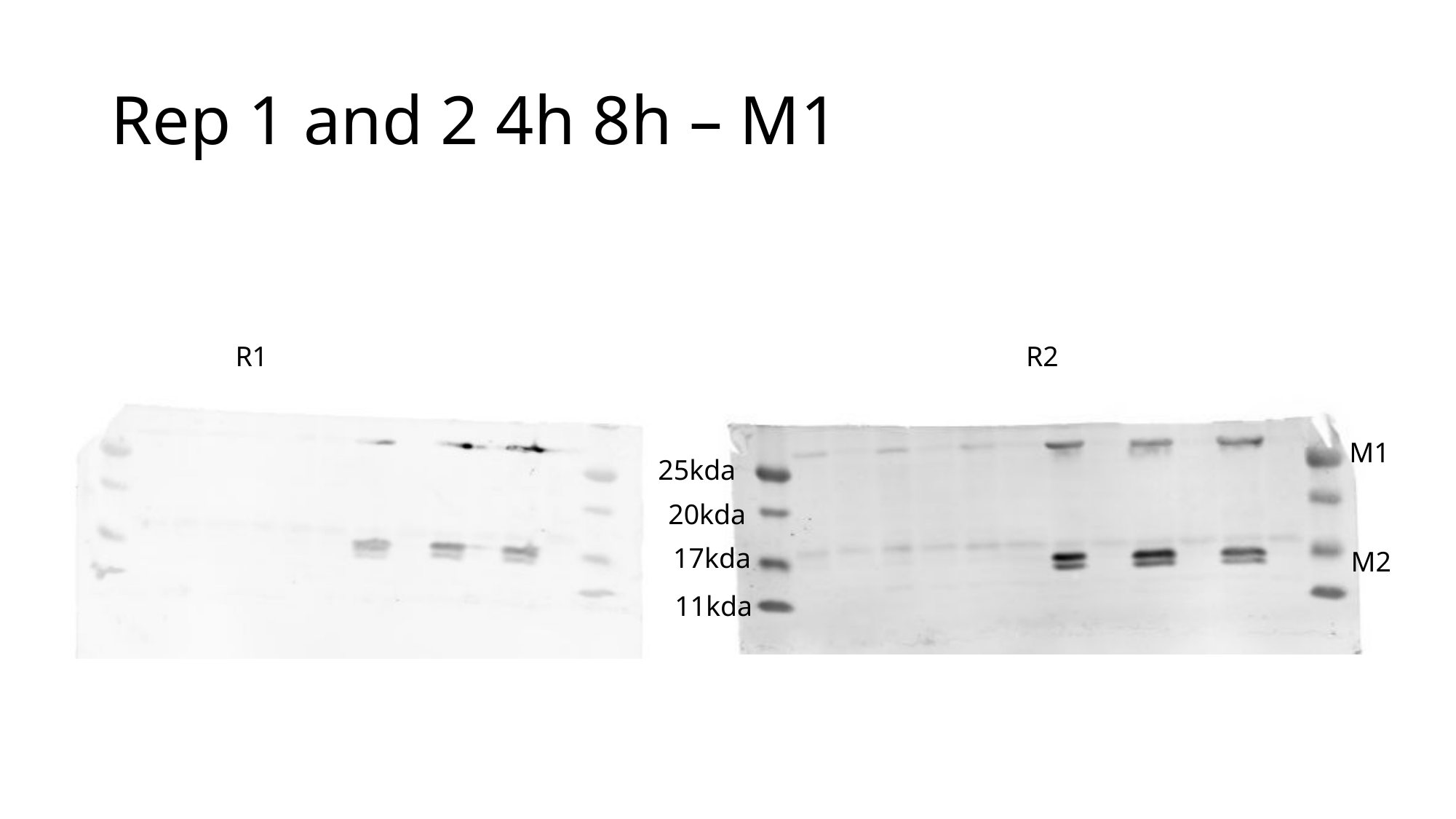

# Rep 1 and 2 4h 8h – M1
R1
R2
M1
25kda
20kda
17kda
M2
11kda

## Slide 9
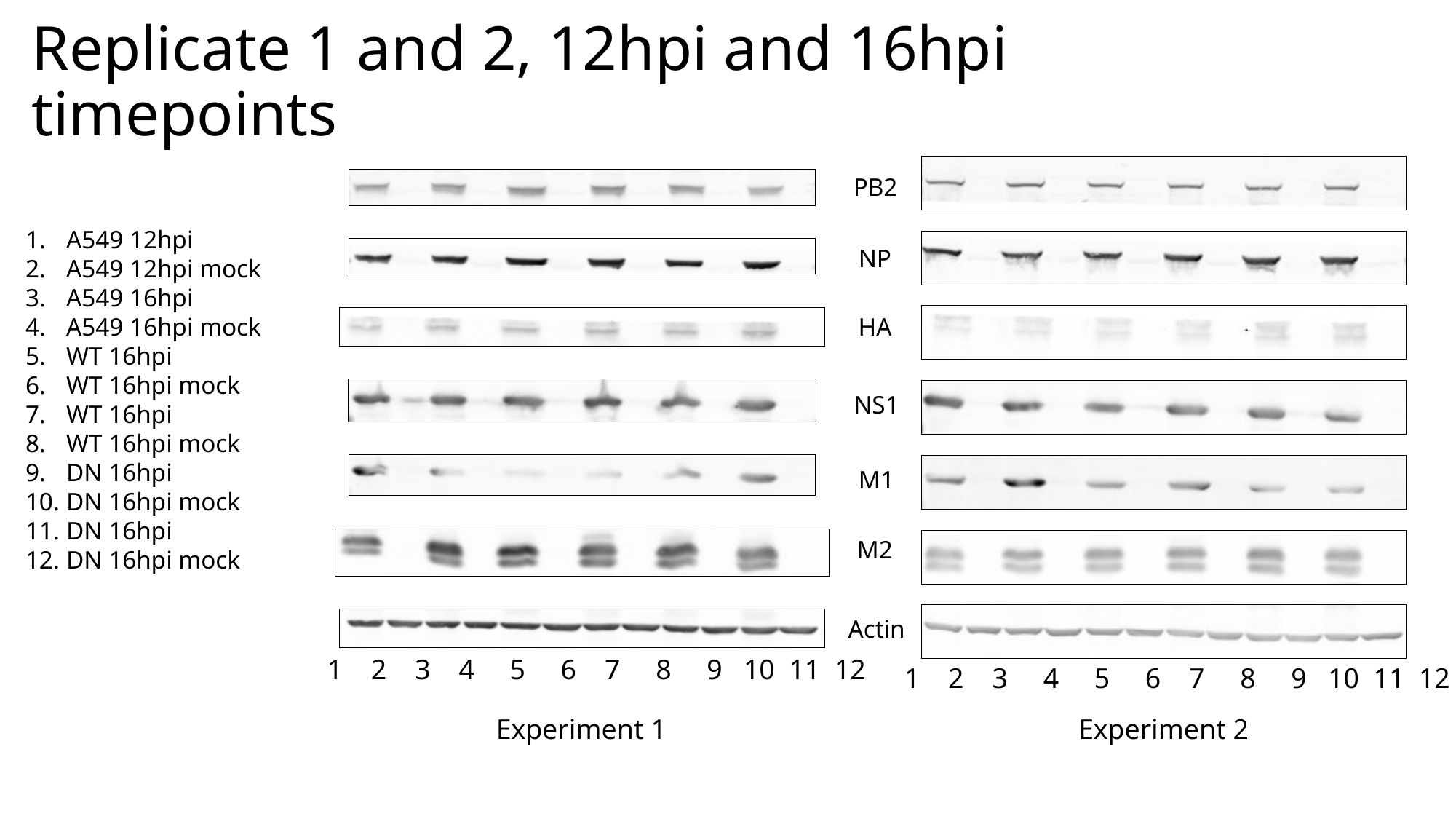

# Replicate 1 and 2, 12hpi and 16hpi timepoints
PB2
A549 12hpi
A549 12hpi mock
A549 16hpi
A549 16hpi mock
WT 16hpi
WT 16hpi mock
WT 16hpi
WT 16hpi mock
DN 16hpi
DN 16hpi mock
DN 16hpi
DN 16hpi mock
NP
HA
NS1
M1
M2
Actin
 1 2 3 4 5 6 7 8 9 10 11 12
 1 2 3 4 5 6 7 8 9 10 11 12
Experiment 1
Experiment 2

## Slide 10
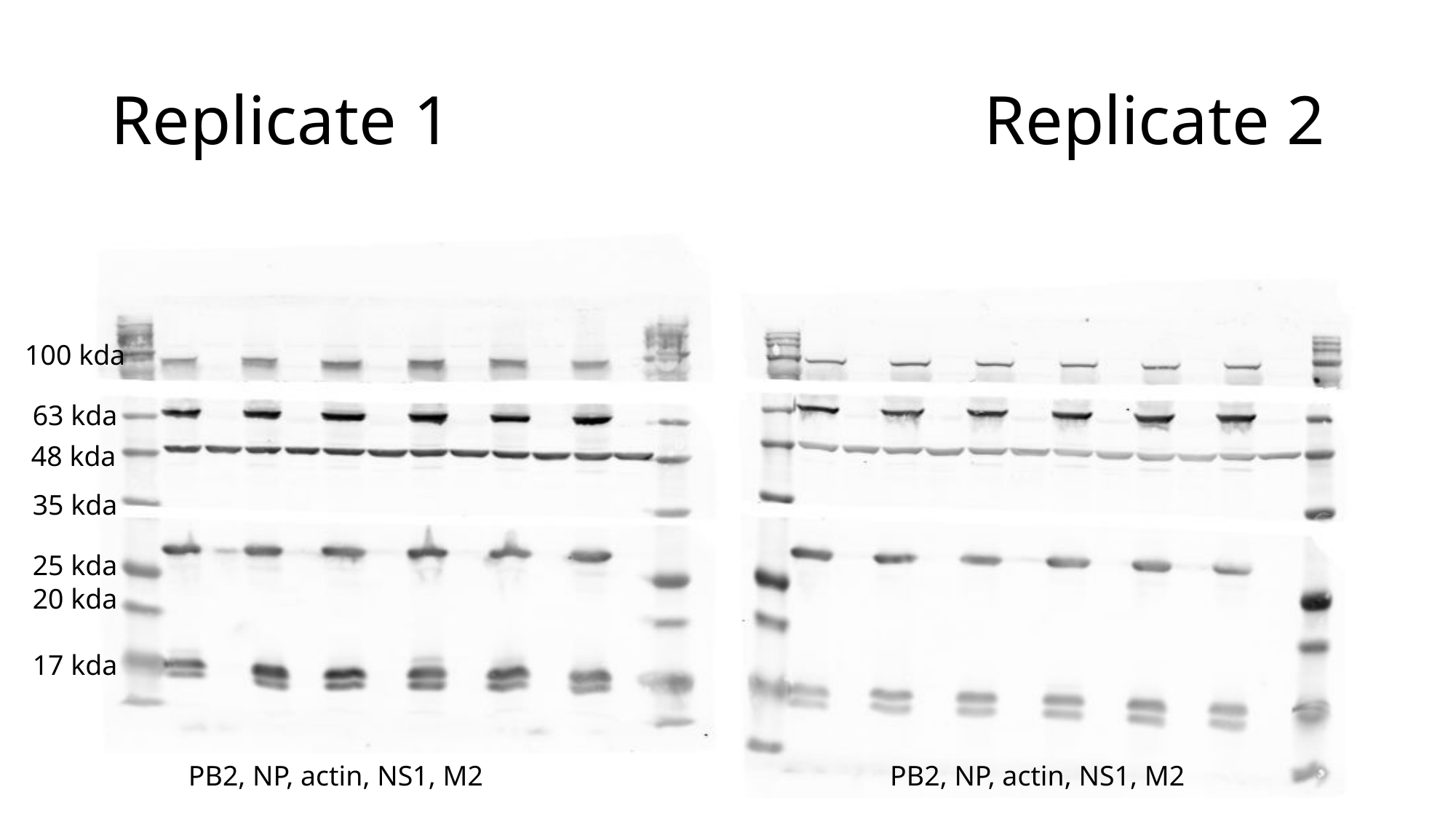

# Replicate 1					Replicate 2
100 kda
63 kda
48 kda
35 kda
25 kda
20 kda
17 kda
PB2, NP, actin, NS1, M2
PB2, NP, actin, NS1, M2

## Slide 11
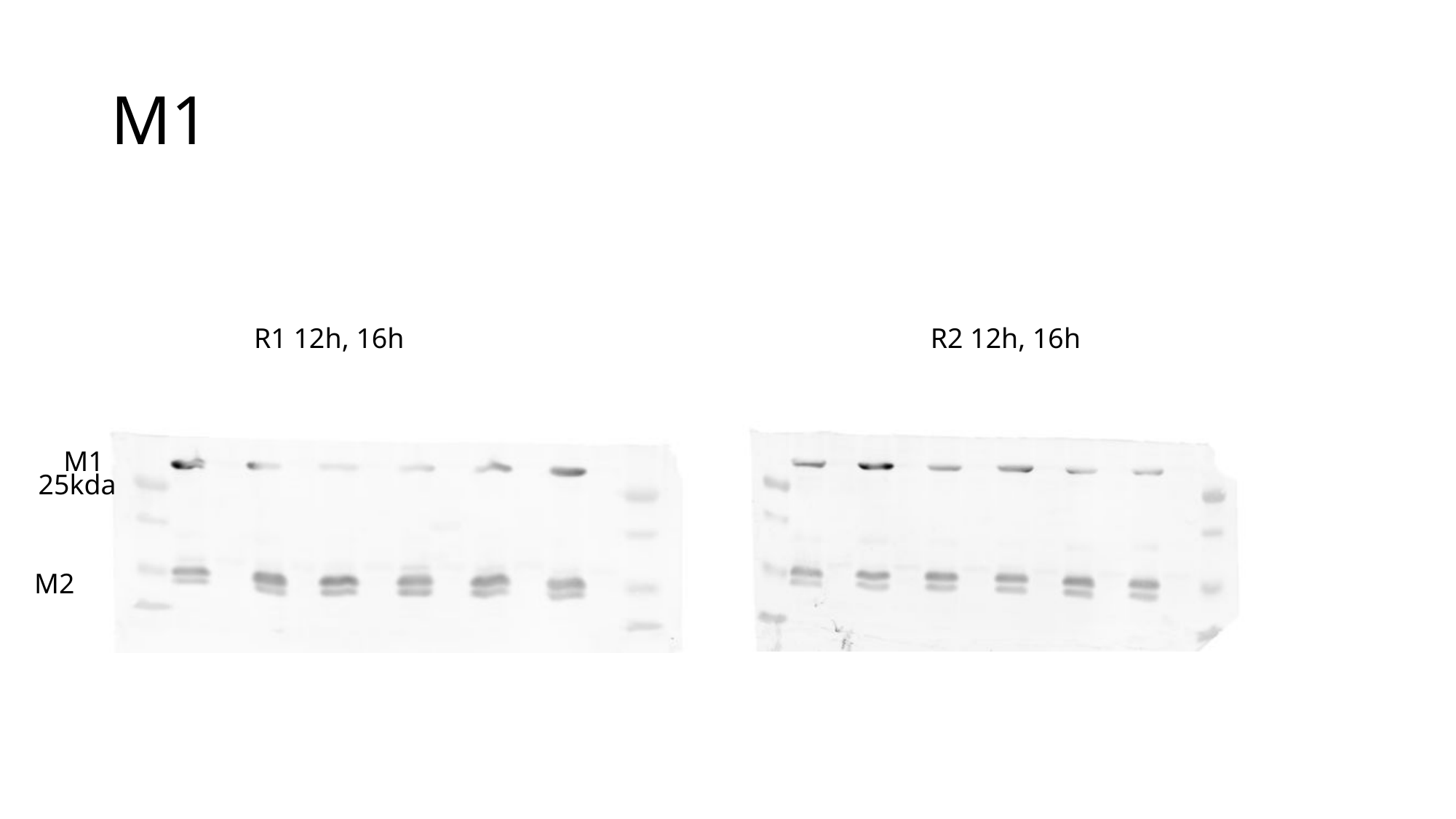

# M1
R1 12h, 16h
R2 12h, 16h
M1
25kda
M2

## Slide 12
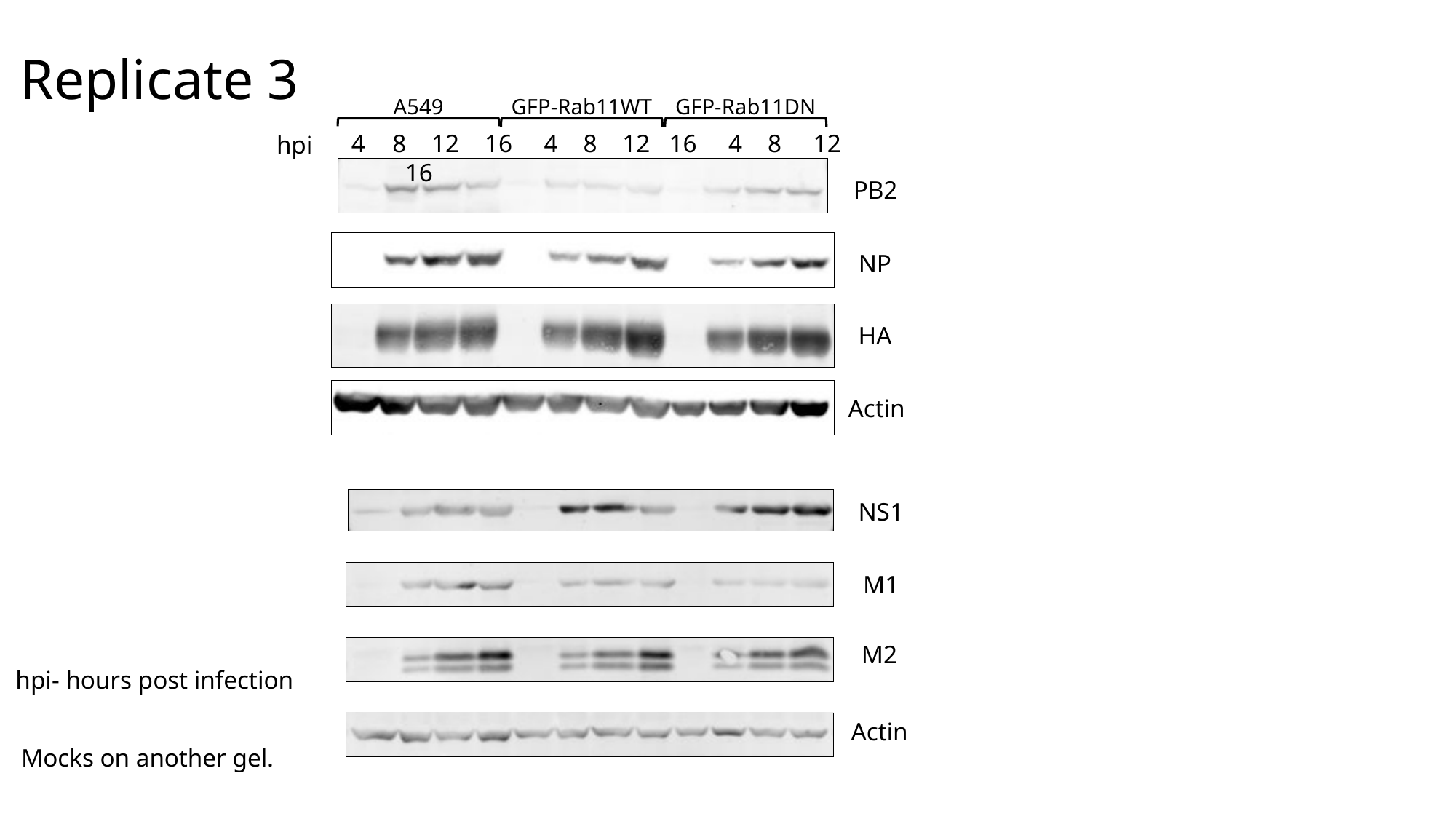

# Replicate 3
A549
GFP-Rab11WT
GFP-Rab11DN
8 12 16 4 8 12 16 4 8 12 16
hpi
PB2
NP
HA
Actin
NS1
M1
M2
hpi- hours post infection
Actin
Mocks on another gel.

## Slide 13
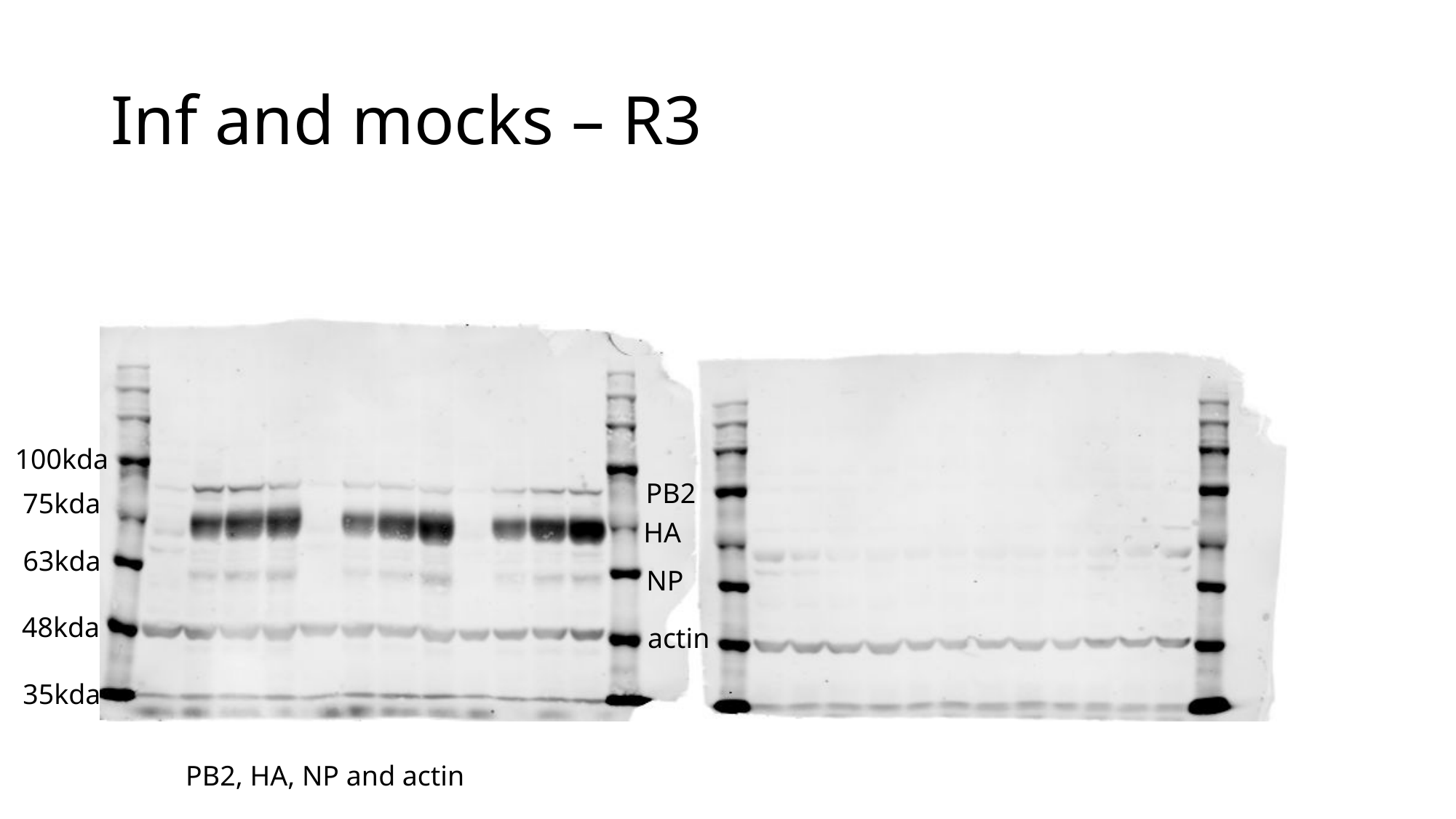

# Inf and mocks – R3
100kda
PB2
75kda
HA
63kda
NP
48kda
actin
35kda
PB2, HA, NP and actin

## Slide 14
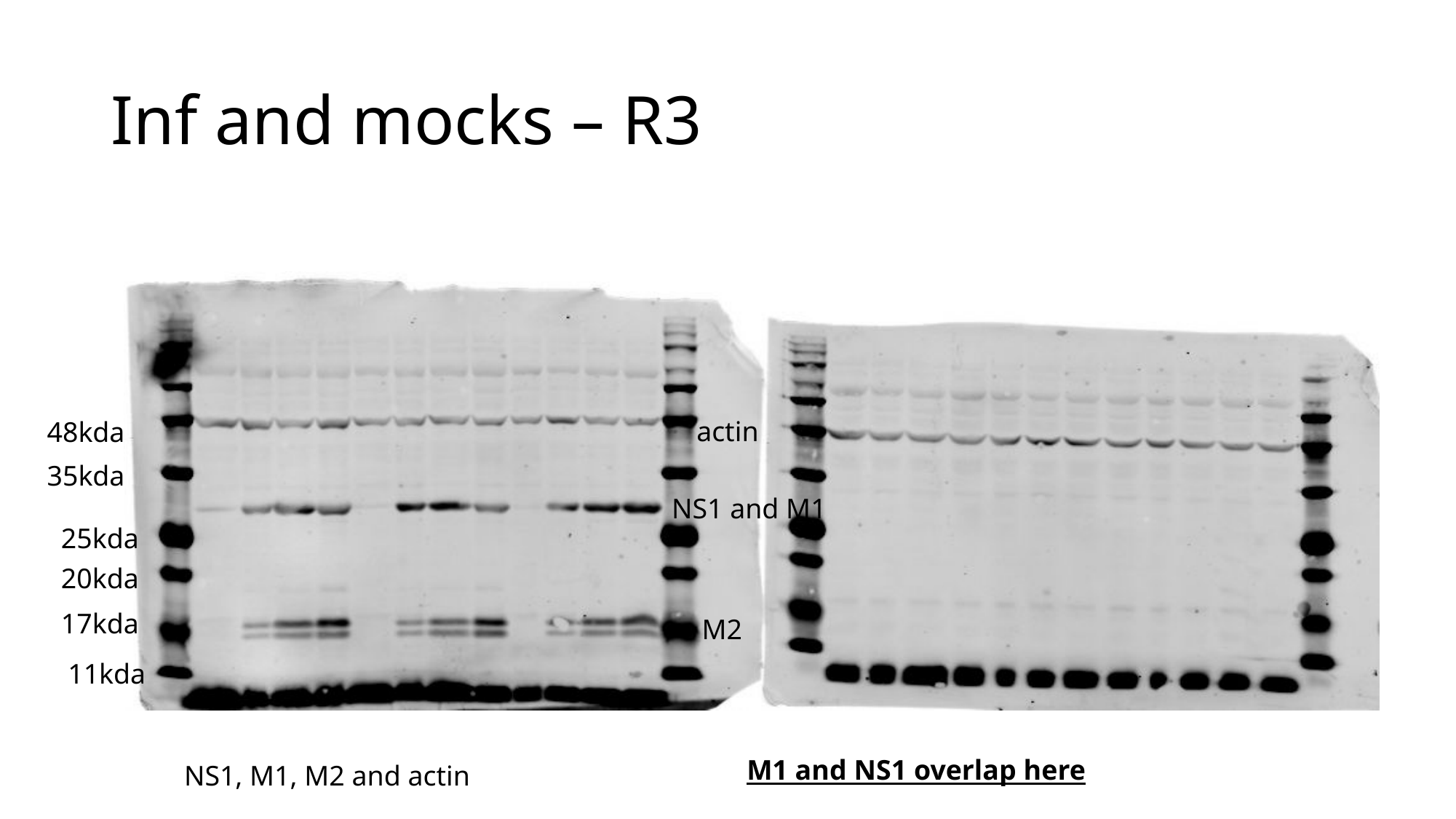

# Inf and mocks – R3
actin
48kda
35kda
NS1 and M1
25kda
20kda
17kda
M2
11kda
M1 and NS1 overlap here
NS1, M1, M2 and actin

## Slide 15
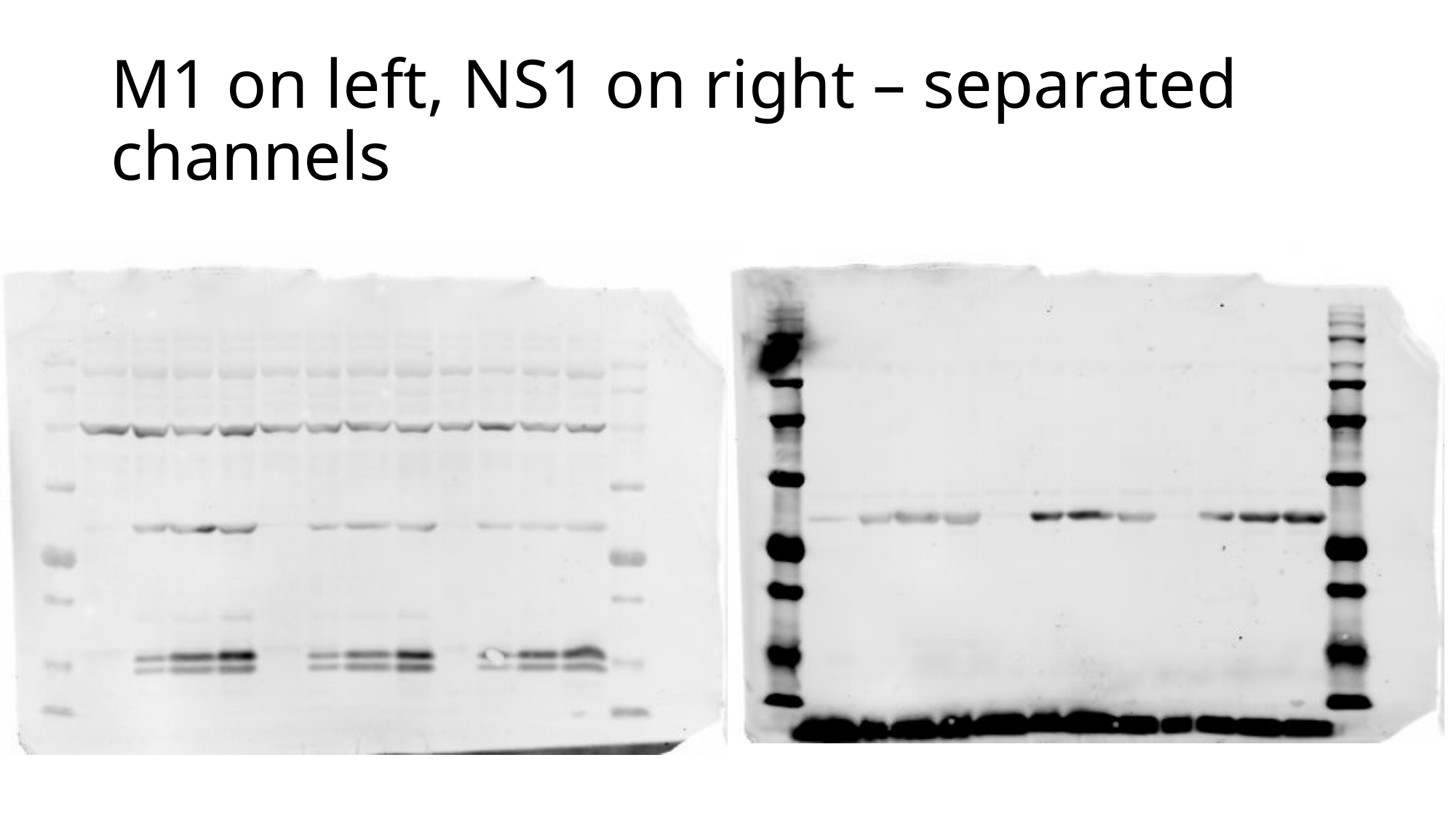

# M1 on left, NS1 on right – separated channels

## Slide 16
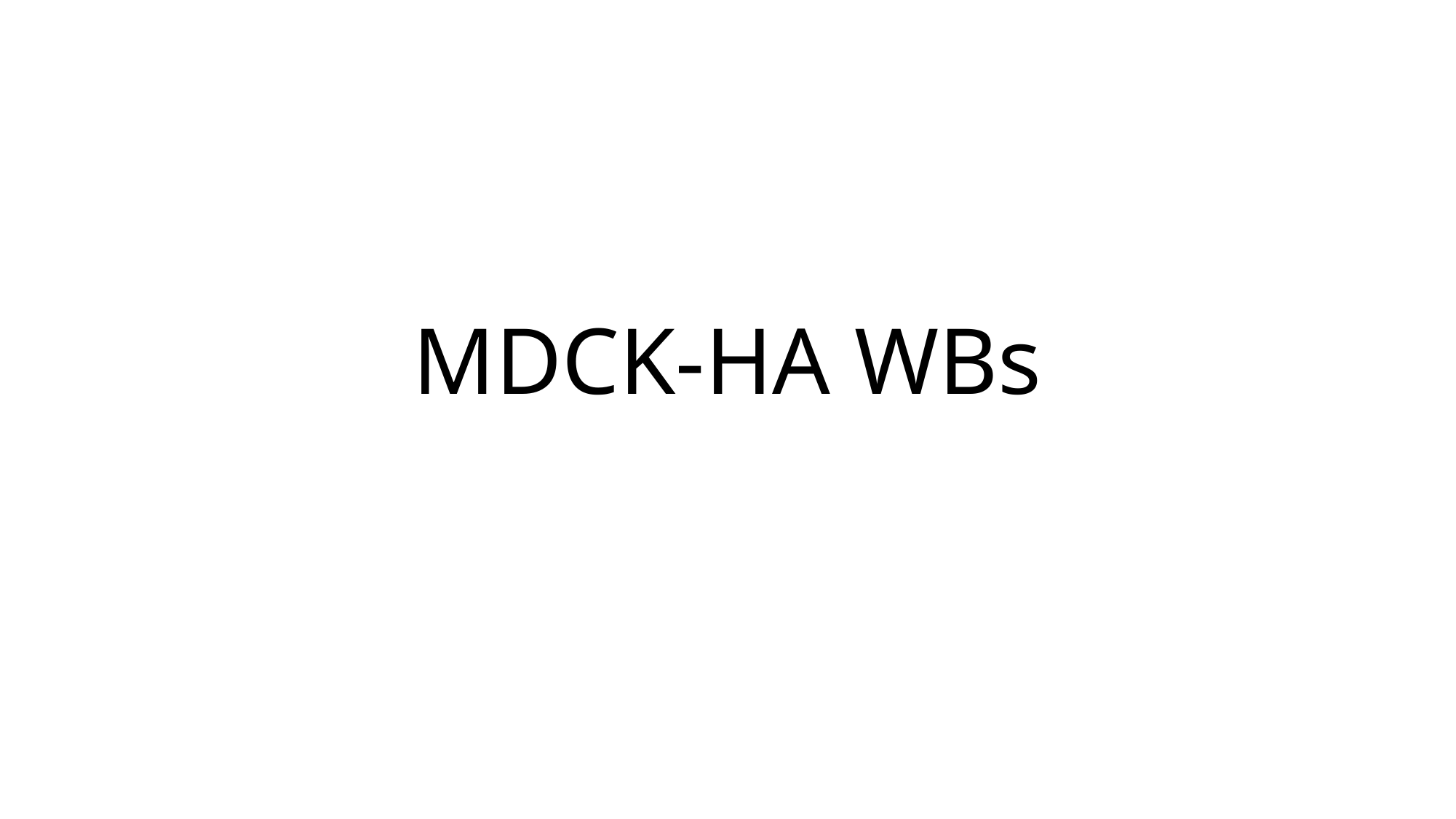

# MDCK-HA WBs

## Slide 17
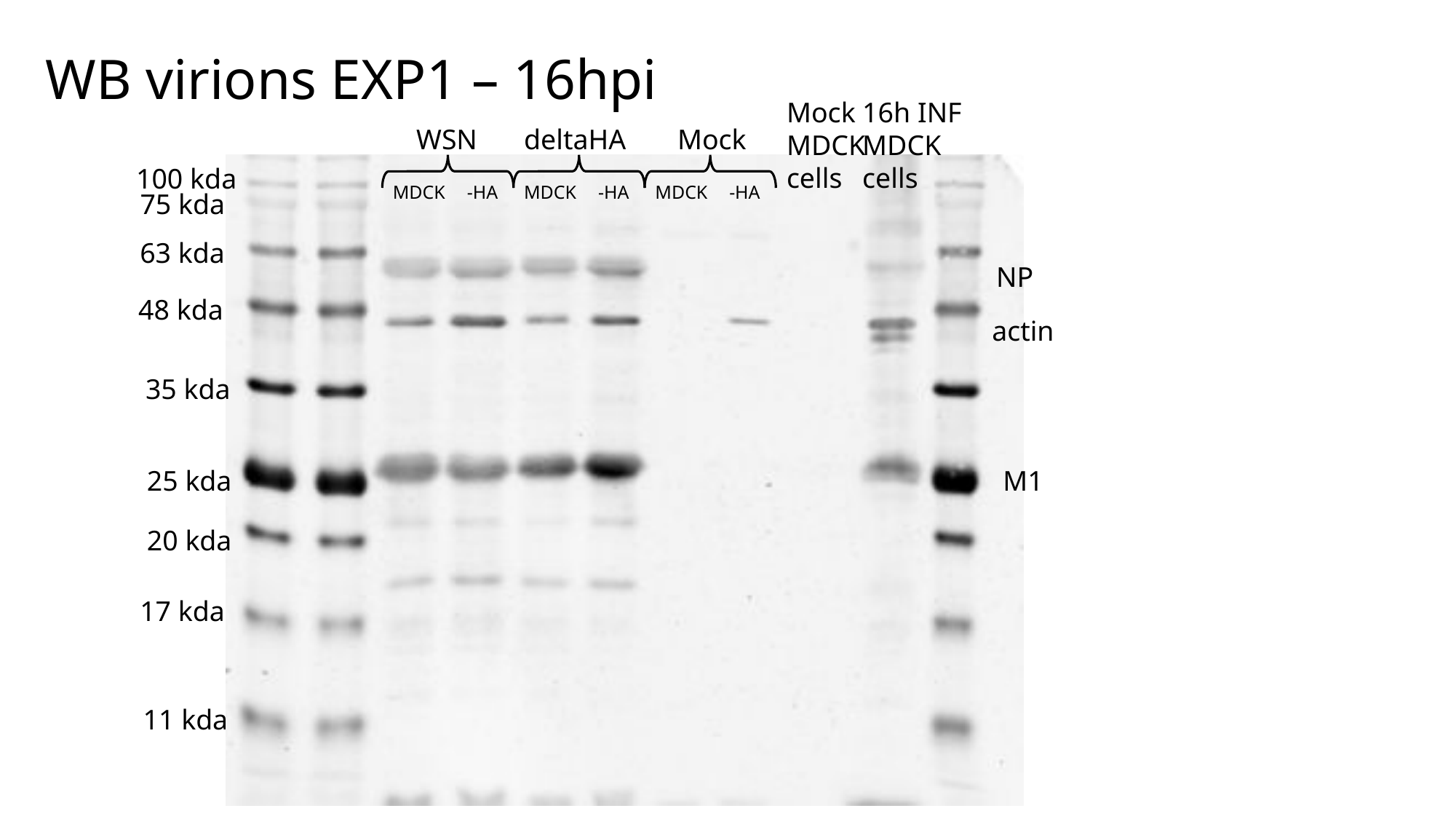

WB virions EXP1 – 16hpi
Mock
MDCK
cells
16h INF
MDCK
cells
WSN
deltaHA
Mock
100 kda
MDCK
-HA
MDCK
-HA
MDCK
-HA
75 kda
63 kda
NP
48 kda
actin
35 kda
25 kda
M1
20 kda
17 kda
11 kda

## Slide 18
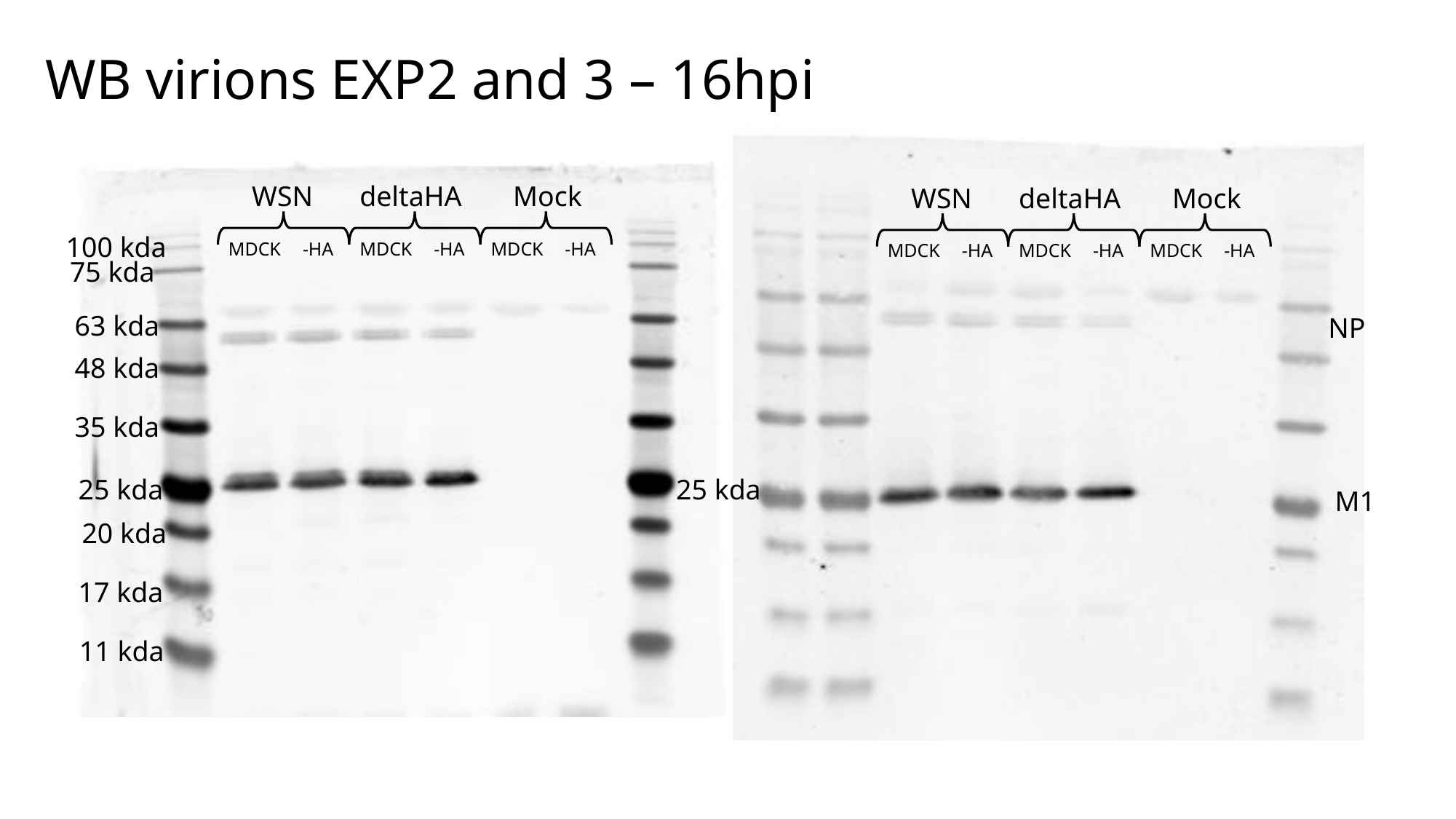

WB virions EXP2 and 3 – 16hpi
WSN
deltaHA
Mock
WSN
deltaHA
Mock
100 kda
MDCK
-HA
MDCK
-HA
MDCK
-HA
MDCK
-HA
MDCK
-HA
MDCK
-HA
75 kda
63 kda
NP
48 kda
35 kda
25 kda
25 kda
M1
20 kda
17 kda
11 kda
