# Supplementary figures and images for: Visualizing influenza A virus assembly by in situ cryo-electron tomography
Source: Nat Commun. 2025 Oct 23;16:9394. doi: 10.1038/s41467-025-65117-z (PMC12550032; doi:10.1038/s41467-025-65117-z)

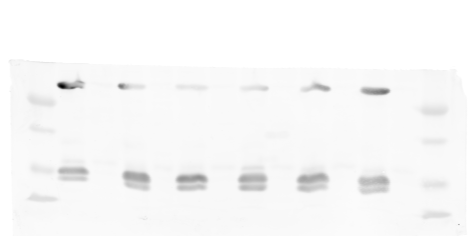

Supplement: Supplementary file 13 — Source Data [file 41467_2025_65117_MOESM13_ESM.zip › western_blots_uncropped/sfig_05_c_PB2_HA_NA_NP_NS1_M1_M2_WB_timeseries/R1R2_A549lines/12h_16h/Chlanda_exp1_12hpi_16hpi_M1/0001719_01_800.png]

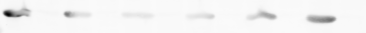

Supplement: Supplementary file 13 — Source Data [file 41467_2025_65117_MOESM13_ESM.zip › western_blots_uncropped/sfig_05_c_PB2_HA_NA_NP_NS1_M1_M2_WB_timeseries/R1R2_A549lines/12h_16h/Chlanda_exp1_12hpi_16hpi_M1/0001719_01_800_M1-1.png]

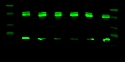

Supplement: Supplementary file 13 — Source Data [file 41467_2025_65117_MOESM13_ESM.zip › western_blots_uncropped/sfig_05_c_PB2_HA_NA_NP_NS1_M1_M2_WB_timeseries/R1R2_A549lines/12h_16h/Chlanda_exp1_12hpi_16hpi_M1/0001719_01_TH.jpg]

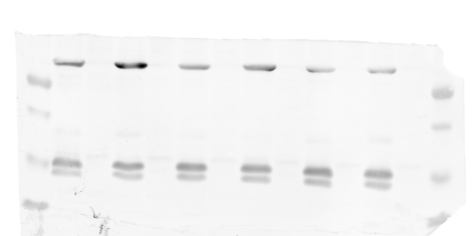

Supplement: Supplementary file 13 — Source Data [file 41467_2025_65117_MOESM13_ESM.zip › western_blots_uncropped/sfig_05_c_PB2_HA_NA_NP_NS1_M1_M2_WB_timeseries/R1R2_A549lines/12h_16h/Chlanda_exp2_12hpi_16hpi_M1/0001721_01_800.png]

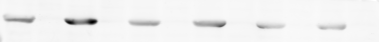

Supplement: Supplementary file 13 — Source Data [file 41467_2025_65117_MOESM13_ESM.zip › western_blots_uncropped/sfig_05_c_PB2_HA_NA_NP_NS1_M1_M2_WB_timeseries/R1R2_A549lines/12h_16h/Chlanda_exp2_12hpi_16hpi_M1/0001721_01_800_M1-1.png]

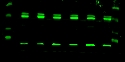

Supplement: Supplementary file 13 — Source Data [file 41467_2025_65117_MOESM13_ESM.zip › western_blots_uncropped/sfig_05_c_PB2_HA_NA_NP_NS1_M1_M2_WB_timeseries/R1R2_A549lines/12h_16h/Chlanda_exp2_12hpi_16hpi_M1/0001721_01_TH.jpg]

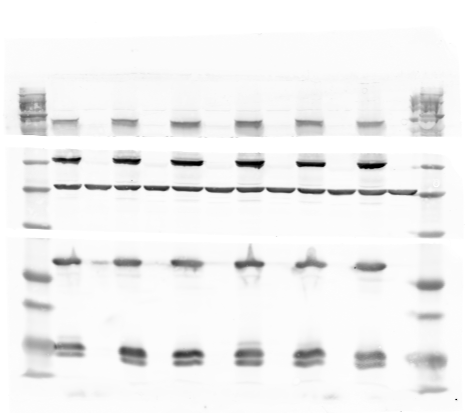

Supplement: Supplementary file 13 — Source Data [file 41467_2025_65117_MOESM13_ESM.zip › western_blots_uncropped/sfig_05_c_PB2_HA_NA_NP_NS1_M1_M2_WB_timeseries/R1R2_A549lines/12h_16h/R1_12h16h_PB2_NP_actin_NS1_M2.png]

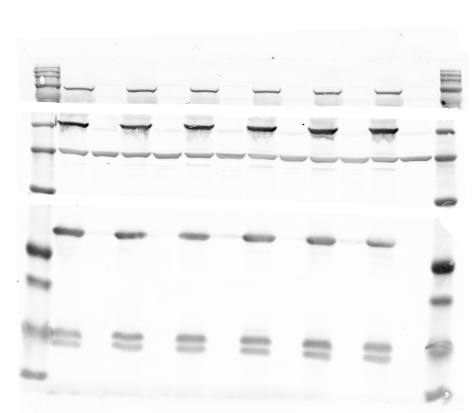

Supplement: Supplementary file 13 — Source Data [file 41467_2025_65117_MOESM13_ESM.zip › western_blots_uncropped/sfig_05_c_PB2_HA_NA_NP_NS1_M1_M2_WB_timeseries/R1R2_A549lines/12h_16h/R2_12h16h_PB2_NP_actin_NS1_M2.png]

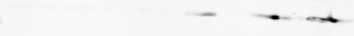

Supplement: Supplementary file 13 — Source Data [file 41467_2025_65117_MOESM13_ESM.zip › western_blots_uncropped/sfig_05_c_PB2_HA_NA_NP_NS1_M1_M2_WB_timeseries/R1R2_A549lines/4h_8h/Chlanda_exp1_4hpi_8hpi_M1/0001718_01_800-1_M1.png]

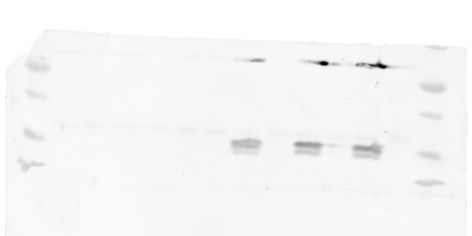

Supplement: Supplementary file 13 — Source Data [file 41467_2025_65117_MOESM13_ESM.zip › western_blots_uncropped/sfig_05_c_PB2_HA_NA_NP_NS1_M1_M2_WB_timeseries/R1R2_A549lines/4h_8h/Chlanda_exp1_4hpi_8hpi_M1/0001718_01_800.png]

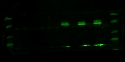

Supplement: Supplementary file 13 — Source Data [file 41467_2025_65117_MOESM13_ESM.zip › western_blots_uncropped/sfig_05_c_PB2_HA_NA_NP_NS1_M1_M2_WB_timeseries/R1R2_A549lines/4h_8h/Chlanda_exp1_4hpi_8hpi_M1/0001718_01_TH.jpg]

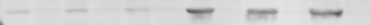

Supplement: Supplementary file 13 — Source Data [file 41467_2025_65117_MOESM13_ESM.zip › western_blots_uncropped/sfig_05_c_PB2_HA_NA_NP_NS1_M1_M2_WB_timeseries/R1R2_A549lines/4h_8h/Chlanda_exp2_4hpi_8hpi_M1/0001720_01_800-1_M1.png]

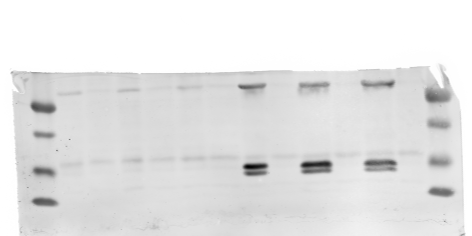

Supplement: Supplementary file 13 — Source Data [file 41467_2025_65117_MOESM13_ESM.zip › western_blots_uncropped/sfig_05_c_PB2_HA_NA_NP_NS1_M1_M2_WB_timeseries/R1R2_A549lines/4h_8h/Chlanda_exp2_4hpi_8hpi_M1/0001720_01_800.png]

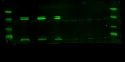

Supplement: Supplementary file 13 — Source Data [file 41467_2025_65117_MOESM13_ESM.zip › western_blots_uncropped/sfig_05_c_PB2_HA_NA_NP_NS1_M1_M2_WB_timeseries/R1R2_A549lines/4h_8h/Chlanda_exp2_4hpi_8hpi_M1/0001720_01_TH.jpg]

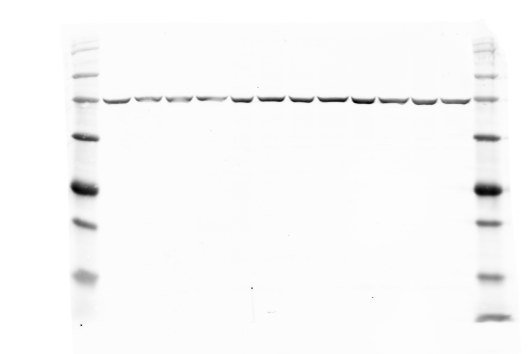

Supplement: Supplementary file 13 — Source Data [file 41467_2025_65117_MOESM13_ESM.zip › western_blots_uncropped/sfig_05_c_PB2_HA_NA_NP_NS1_M1_M2_WB_timeseries/R1R2_A549lines/4h_8h/R1R24h8h_HA_actin_Mock.png]

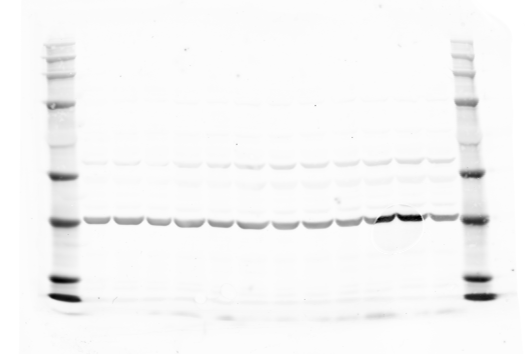

Supplement: Supplementary file 13 — Source Data [file 41467_2025_65117_MOESM13_ESM.zip › western_blots_uncropped/sfig_05_c_PB2_HA_NA_NP_NS1_M1_M2_WB_timeseries/R1R2_A549lines/4h_8h/R1R24h8h_mocks_PB2_NP_actin.png]

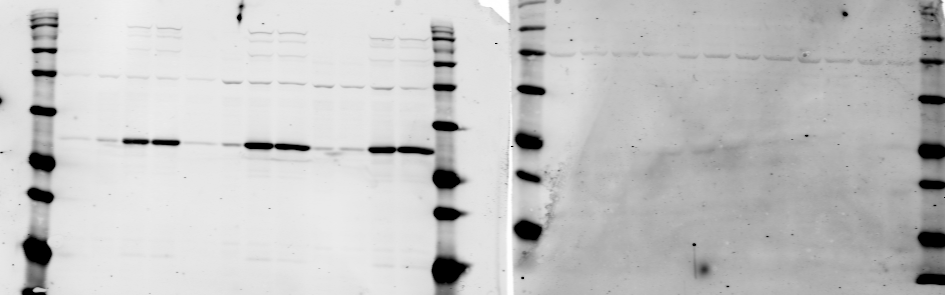

Supplement: Supplementary file 13 — Source Data [file 41467_2025_65117_MOESM13_ESM.zip › western_blots_uncropped/sfig_05_c_PB2_HA_NA_NP_NS1_M1_M2_WB_timeseries/R1R2_A549lines/4h_8h/R1R2_4h8h_NS1.png]

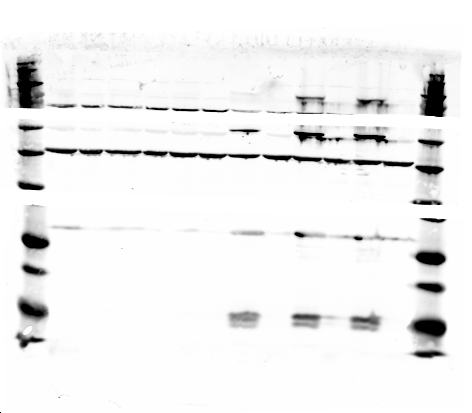

Supplement: Supplementary file 13 — Source Data [file 41467_2025_65117_MOESM13_ESM.zip › western_blots_uncropped/sfig_05_c_PB2_HA_NA_NP_NS1_M1_M2_WB_timeseries/R1R2_A549lines/4h_8h/R1_M2_actin.png]

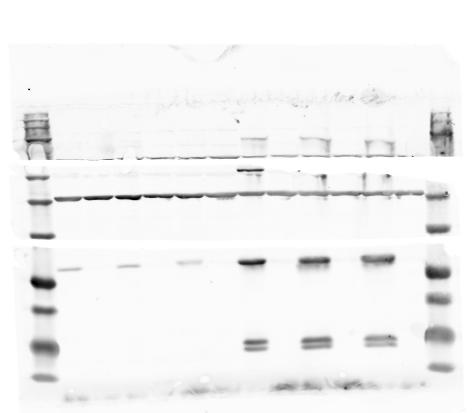

Supplement: Supplementary file 13 — Source Data [file 41467_2025_65117_MOESM13_ESM.zip › western_blots_uncropped/sfig_05_c_PB2_HA_NA_NP_NS1_M1_M2_WB_timeseries/R1R2_A549lines/4h_8h/R2_M2_actin.png]
